# Occurrence of PFAS in municipal drinking water: a participatory case study in London, UK

**DOI:** 10.1039/d6va00076b

**Published:** 2026-05-12

**Authors:** Alexandra K. Richardson, Wei-Han Tien, Charlotte I. Z. O'Hern, William Francis, Sarah Dack, Leon P. Barron, Frédéric B. Píel

**Affiliations:** a Department of Epidemiology & Biostatistics, School of Public Health, Imperial College London London UK a.richardson@imperial.ac.uk; b MRC Centre for Environment & Health, Environmental Research Group, School of Public Health, Imperial College London London UK; c NIHR Health Protection Research Unit in Environmental Exposures & Health, School of Public Health, Imperial College London London UK; d Department of Analytical, Environmental and Forensic Sciences, Institute of Pharmaceutical Science, King's College London London UK; e UK Health Security Agency London UK

## Abstract

Globally, per- and poly-fluoroalkyl substances (PFAS) contamination has been reported in numerous environmental matrices, and there is a growing body of evidence that links PFAS exposure to adverse health effects. Consuming contaminated drinking water is potentially one of the most common routes of human exposure from these compounds. The spatial and temporal variability of 38 PFAS in 210 household tap water and public water fountains were assessed using a participatory sampling campaign in London, UK. The performance of commercially available water filters to remove PFAS was also assessed. Individual PFAS concentrations ranged from 0.6 ± 0.1 ng L^−1^ (PFBS) to 9.1 ± 0.2 ng L^−1^ (PFOS), and total PFAS concentrations ranged from 3 ng L^−1^ to 41 ng L^−1^ (mean = 18 ± 8 ng L^−1^, median = 18 ng L^−1^). Overall, 100% (*n* = 210) of all tap drinking water samples tested were within the lowest action threshold currently in place for England (<10 ng L^−1^ for individual PFAS), and all samples were below the threshold for total PFAS (<100 ng L^−1^). The daily concentration of PFAS did not substantially vary over the course of a month in three homes tested intensively. The risk to humans posed by four specific PFAS (PFOS, PFOA, PFNA, and PFHxS) in London drinking water was below the weekly tolerable intake established by the European Food Standards Authority (EFSA). Five water filters tested removed at least 85% of all PFAS studied in spiked (50 ng L^−1^) water samples, therefore providing an effective way to reduce concentrations in regions where such contamination is of greater concern and/or where PFAS are not routinely monitored. Our findings provide reassuring evidence about the quality of municipal drinking water in London and the UK as a whole when considering official measurements made at treatment plants. We also provide benchmark risk assessment data for the future and information to concerned citizens about the quality of tap drinking water.

## Abstract 2

Globally, per- and poly-fluoroalkyl substances (PFAS) contamination has been reported in numerous environmental matrices, but data on their occurrence in drinking water within UK households remains limited.

## Graphical abstract

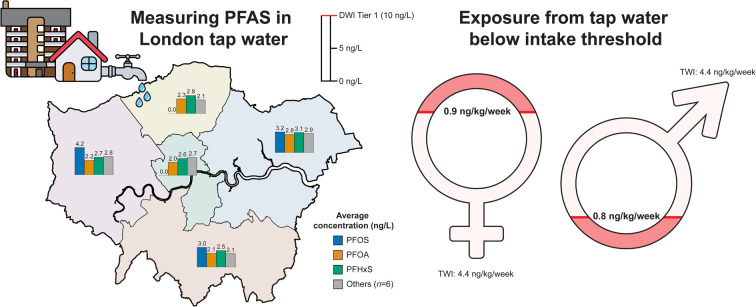

**Environmental significance**

There is growing evidence that PFAS contamination is widespread, and there are increasing concerns about the risks these substances may pose to human health. Drinking contaminated tap water likely represents one of the most common routes of PFAS exposure. As such, it is important to understand and assess the risks associated with this pathway of exposure. This study evaluated the concentration of PFAS in 210 samples of tap water in London, England, assessed the risk to human health, and investigated the efficiency of water filter jugs to remove PFAS from contaminated tap water. The concentration of PFAS in all tap water samples was below the lowest action threshold for England, and the risk to human health was estimated to be low.

## 1 Introduction

The importance of consuming water cannot be understated; it is vital for human health and plays an important role in multiple metabolic processes and maintaining homeostasis.^1^ Access to clean and wholesome drinking water is a cornerstone of public health, critical for disease prevention and overall well-being. Aside from the acute effects caused by exposure to bacteria, viruses, and parasites *via* drinking water sources, the long-term effects of exposure to trace concentrations of chemical contaminants are a growing threat to human health.^2^ Contaminants of concern in drinking water recognised by the World Health Organisation (WHO) include inorganic compounds (*e.g.*, ammonia and nitrate/nitrite), metals (*e.g.*, arsenic and lead), pesticides (*e.g.*, atrazine and permethrin), and legacy manufacturing by-products (*e.g.*, per- and polyfluoroalkyl substances (PFAS) and toluene).^3^

Often termed “forever chemicals”, PFAS are a large group of over 4000 compounds.^4^ They were first synthesised in the 1940s and are broadly defined as any aliphatic substances containing a perfluoroalkyl moiety either as a methyl group (–CF_3_) or methylene group (–CF_2_–).^4–6^ The chemical properties conferred by the moiety (*e.g.*, chemical/thermal stability, hydrophobicity, and lipophobicity) are extremely useful in a variety of industrial applications and consumer products, leading to the widespread use of PFAS globally.^7,8^ However, the strength and stability of the C–F bonds make PFAS extremely resistant to natural degradation processes, resulting in high environmental persistence and concerns about human health effects from long-term exposure.^9^ A growing body of research has linked PFAS exposure with a variety of health effects, including developmental issues, disruptions in lipid metabolism and the endocrine system, and serious conditions such as cancer, immune system toxicity, liver damage, and reproductive toxicity.^10–14^ As such, several PFAS (namely PFOS, PFOA, PFHxS, and PFNA, see SI Table S1 for full names) are regulated by the United Nations Stockholm Convention and the European Commission Persistent Organic Pollutants (POPs) Regulation.^15–19^ Although further work is needed to fully understand the causal link between PFAS exposure and human health, there is growing public awareness about possible risks. As the predominant route of human exposure to PFAS in the general population is likely to be *via* the consumption of contaminated foods and drinks (>90%)^20^ and 97% of adults in the UK drink tap water,^21^ it is essential to assess the risk associated with this pathway of exposure to PFAS, and to balance this risk in relation to other factors (*e.g.*, dehydration risk, microplastic exposure, *etc.*).

Globally, investigations of PFAS contamination of municipal tap water have been conducted for over 250 different compounds in 30 countries since 2003, with concentrations measured ranging from undetectable levels to 519 ng L^−1^ (SI Table S2). However, the number of PFAS tested, the sample preparation techniques, and the method sensitivity varies substantially between studies. For example, Alghamdi *et al.* utilised solid-phase extraction to achieve sub nanogram PFAS quantification in tap water samples collected from households in Victoria, Australia,^22^ while Skaggs & Logue developed a novel ice concentration technique (ICECLES) to measure trace concentrations of PFAS in drinking water.^23^ However, several studies may have sampled areas where concerns were raised (*i.e.*, bias towards high concentrations), rather than through random or representative sampling.

As a first step to protect human health, guidelines for acceptable levels of PFAS in tap water have been established in multiple countries. Some of the most stringent guidelines were set in 2022 by the US Environmental Protection Agency (EPA) for PFOS and PFOA with a maximum contaminant level (MCL) of 4 ng L^−1^ for each.^24,25^ In Europe, the revised Drinking Water Directive 2020, requires that: (i) the total concentration of 20 specific PFAS compounds should not exceed 100 ng L^−1^, and (ii) the total of all PFAS present should be limited to 500 ng L^−1^.^26^ In 2020, the European Food Standards Authority (EFSA) updated the safety threshold for the tolerable weekly intake (TWI) for the sum of four PFAS compounds (PFOA, PFOS, PFNA, and PFHxS) to 4.4 ng per kilogram (ng kg^−1^) of body weight per week.^27^ Towards the end of 2025, the monitoring and reporting obligations for 24 PFAS for surface water were being considered as part of an updated EU Water Framework and Drinking Water Directives.^28^ Since 2023, the European Chemicals Agency's (ECHA) Risk Assessment Committee (RAC) has been considering extensive restrictions on PFAS, with a formal option to be adopted in early 2026.

In England and Wales, the regulation of PFAS compounds in drinking water is the responsibility of the Drinking Water Inspectorate (DWI). It has adopted a risk-based approach for 48 PFAS compounds since 2021.^29^ The remit of the DWI is to provide independent reassurance that water supplies from the water companies in England and Wales is wholesome, and that drinking water quality is acceptable to consumers. Under DWI guidelines, PFAS concentrations below 10 ng L^−1^ are considered low risk, but with ongoing monitoring and hazard assessment to determine if further action needs to be taken by the drinking water provider in the future. For PFAS concentrations greater than 10 ng L^−1^, proactive and systematic risk reduction strategies are employed to prevent any increase in concentrations. Where PFAS concentrations meet or exceed 100 ng L^−1^, this is considered to breach wholesomeness standards and constitutes a potential hazard to human health. In this scenario, the water company must inform consumers, DWI, UK Health Security Agency (UKHSA), and local health authorities, and immediate action must be taken to remediate the water supply.^30^ To align England's PFAS guidance more closely with international practices, the Royal Society of Chemistry (RSC) proposed to reduce the action standard from 100 ng L^−1^ to 10 ng L^−1^ for individual PFAS and to implement an aggregate PFAS limit of 100 ng L^−1^ for the 48 PFAS compounds that were currently monitored.^31^ The DWI extended their guidelines to adopt the cumulative sum proposed by the RSC, which was implemented from the 1^st^ of January 2025.^30^ In February 2026, the Department for Environment, Food & Rural Affairs (DEFRA) released their plan on how the UK government will address the risks posed by PFAS, with an action to consult on the introduction of an enforceable statutory limit on PFAS concentrations in the public water supply later in the year.^32^

There is limited publicly held data on PFAS concentrations generally in the UK and for English drinking water, particularly at the point of consumption, which is what matters from a public health perspective. Here, we aimed to better understand human exposure to PFAS *via* municipal tap water in Greater London, England. London is the largest city in England with a population of over 8.9 million people (approximately 13% of the UK population). This was achieved by: (i) studying the temporal and spatial variability of PFAS in municipal tap water and public water fountains across London; (ii) estimating the health risks associated with drinking tap water in London; (iii) comparing our London data with other PFAS measurements from across England; and (iv) assessing the removal of PFAS from tap water *via* commercially available portable water filtration jugs. To the best of our knowledge, this serves as one of the first comprehensive studies of PFAS in drinking water at the point of consumption and within UK homes and water fountains, as well as a quantitative assessment of options for filter removal at source for other regions impacted by PFAS contamination.

## 2 Materials and methods

### 2.1. Reagents and consumables

All reagents used were at least HPLC-MS grade or higher unless stated otherwise and confirmed to have low PFAS contamination by in-house testing. Methanol (MeOH) and bottled water were obtained from VWR Scientific (Leicestershire, UK) and Fisher Scientific (Leicestershire, UK), respectively. For quantitative targeted analysis, a standard mix of 54 PFAS standards, including 38 native PFAS analytes listed in the DWI guidelines and 16 stable isotopic-labelled internal standards (SIL-IS) with a purity of ≥97% were purchased from Wellington Laboratories (Guelph, CAN) and Chiron (Trondheim, NOR). Refer to SI Table S1 for full details.

### 2.2. Temporal assessment of PFAS concentrations in tap water

To assess daily variability in PFAS concentrations in tap water, kitchen tap samples were collected daily throughout March 2022 from three households located in Greater London, and externally in Berkshire and Oxfordshire counties for comparison (SI Fig. S1). Water samples were collected directly from the tap using 30 mL polypropylene Nalgene bottles that had been pre-rinsed with tap water three times prior to collection. Controls (*n* = 3 to 9 per household) were sporadically collected throughout the sampling period by transferring HPLC-grade bottled water from one bottle to another to detect potential contamination during the sampling process.

### 2.3. Spatial assessment of PFAS concentrations in tap water

To study the geographical variability of PFAS concentrations in London, we adopted a participatory science approach. Sampling kits were sent to volunteer participants for the collection of tap water samples at home (SI Fig. S2). Samples were collected in two phases, from March to April 2024 and June to September 2024 with sampling time and day varying between the households. All tap water samples were returned through the post or stored in the household freezer (−18 °C) before transport of all samples to the laboratory, where they were stored at −20 °C until analysis. In addition, 12 public water fountains across London were sampled on three separate occasions (7^th^ of March, 22^nd^ of March, and 4^th^ of April 2022, SI Fig. S1). Controls were collected at each site on the same day after each sample. Further details on the sampling methods and sample preparation are provided in SI Methods S1 and S2.

London drinking water is typically abstracted from both ground and surface water sources, though the ratio of ground to surface water varies by drinking water company and season. However, the number and location of abstraction sites is restricted information, with many individual sources blended as they enter the drinking water treatment works.

### 2.4. Instrumental analysis

All water samples were analysed for 38 of the 48 native PFAS listed by the DWI (SI Table S1) using a Shimadzu Nexera X2 LC and LCMS-8060 (Shimadzu Corporation, Kyoto, Japan) with a PFAS delay column (SI Methods S3).

### 2.5. Exposure risk assessment

For the risk assessment, daily exposure to PFAS *via* tap water sources was determined as per eqn (1) where [PFAS_i_] represents the concentration of PFAS in each individual water sample (ng L^−1^), IR_H_2_O_ describes the water intake rate of an adult (L per day), and the average body weight (BW ± standard error) of adults in the UK (over 16 years of age): female = 73 ± 0.4 kg, male = 86 ± 0.5 kg.^33^1
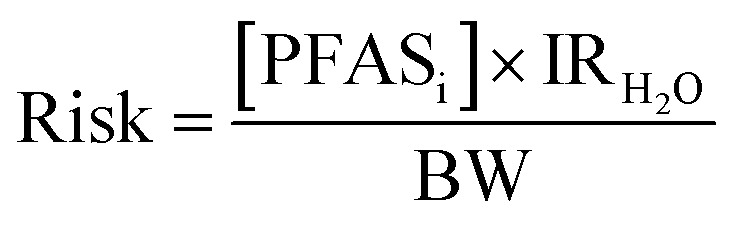


### 2.6. Comparison with national datasets

We compared our PFAS concentrations measured in London with all measurement data freely available from the Environment Agency (EA) Water Quality Monitoring programme through the Department for Environment Food & Rural Affairs (DEFRA) Data Services Platform,^34^ and from the DWI Chief Inspector's report for drinking water in England.^35^ We compared the proportion of measurements in each tier of the DWI assessment system (tier 1: <10 ng L; tier 2: 10–100 ng L; tier 3: >100 ng L^−1^) for all PFAS detected in both London and the rest of England to put our findings in perspective at a national level.

### 2.7. Removal of PFAS using portable filter jugs

To assess portable residential PFAS removal methods from drinking water, five different commercially available water filter jugs were evaluated. Four were specifically designed to remove PFAS from drinking water (a blend of activated carbon (AC) and ion-exchange (IEX) resins). The last filter is a widely used filter for general use (AC only). Triplicates of each jug model and filter were tested. Further details of this assessment are available in SI Methods S4. Each jug was scored based on PFAS removal for all compounds tested. A score of 1 was given for removal rates greater than 95% with an integer penalty for every 5% decrease in efficiency, ranging from scores 1–8. The best-performing filter jug was then used to evaluate the long-term PFAS removal performance of the filter cartridge by continuously filtering over 100 litres of tap water. This represented approximately two months of continuous use with the same filtering cartridge for one adult, based on the daily filtration of 1.5 to 2 L of tap water, in line with the UK National Health Service (NHS) recommended daily water intake.^36^

### 2.8. Statistical analyses

Python (v.3.7.12, Python Software Foundation, DE, USA) packages ‘pandas’ and ‘NumPy’ were used for data handling and analysis and ‘seaborn’ was used for data visualisations.^37–40^ Parametric (Analysis of Variance (ANOVA)) and non-parametric (Kruskal–Wallis Test (KW)) statistical analysis were performed in *R* v.4.4.2 using the ‘dplyr’ and ‘onewaytests’ packages.^41–43^ Data normality and homogeneity of variance were tested using the Levene's test (‘car’ package)^44^ and the Shapiro–Wilk test. If the concentration of a PFAS was below the method's lower limit of quantification (LLOQ), but above the limit of detection (LOD), it was assigned half the LLOQ concentration during statistical analysis as a reasonable approach to minimise any underestimation of total PFAS concentrations.^45,46^ Maps were created using the *R* ‘sf’, ‘rmapshaper’, and ‘ggplot2’ packages^47–49^ using shapefiles sourced from the Office of National Statistics Open Geography Portal,^50^ and the House of Commons Library.^51^

## 3 Results

### 3.1. Temporal assessment of PFAS concentrations in tap water

**Fig. 1:**
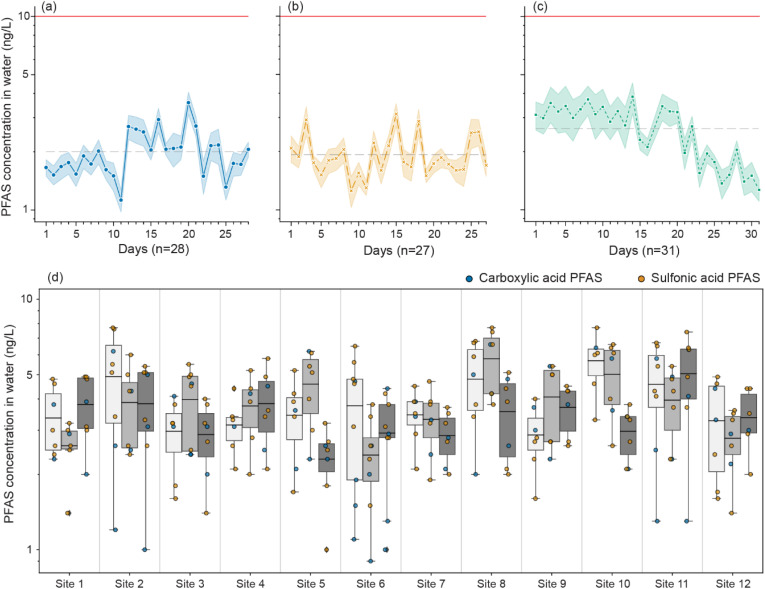
The daily average of PFAS (*n* = 10) concentration in tap water sampled over one month from three households in Greater London (a), Oxfordshire (b), and Berkshire (c). Shaded regions represent standard error, the grey horizontal line represents the average concentration across the whole sampling period, and the red line represents the 10 ng L^−1^ DWI limit. Refer to SI Fig. S3 for total PFAS concentration over the sampling period. (d) Boxplot representing the range of PFAS quantified at each of the 12 drinking water fountains over the three sample collections (light grey = 7^th^ of March 2022, grey = 22^nd^ of March 2022, dark grey = 7^th^ of April 2022). Coloured dots indicate the functional group of the PFAS quantified. Refer to SI Fig. S4 for individual PFAS compounds.

In total, 97 tap water samples (3686 measurements) were collected daily from three households over 31 days in 2022, representing two different drinking water suppliers (Thames Water Ltd and SES Water Ltd). Both suppliers source drinking water from groundwater (Thames Water Ltd: 30–40% and SES Water: 85%) and surface water reservoirs (Thames Water Ltd: 70–60% and SES Water: 15%).^52,53^ Out of the 38 PFAS studied, ten compounds (PFBA, PFBS, PFecHS, PFHpA, PFHxA, PFHxS, PFOA, PFOS, PFPeA, PFPeS) were quantifiable, ranging from 0.7 ± 0.3 ng L^−1^ (PFBA) to 9.1 ± 0.2 ng L^−1^ (PFOS). Across the three households, the average daily individual concentration of PFAS was 2.0 ± 0.9, 1.9 ± 0.8, and 2.7 ± 2.0 ng L^−1^ (Fig. 1). Two PFAS compounds were present in every sample: PFBS and PFHxA. Though there were daily fluctuations in PFAS concentrations within the same household, there was no statistical difference between the sequential samples collected daily across all households (*p* > 0.05, KW test). In two of the three households, PFOS and PFOA did not exceed the US EPA MCL during the sampling period (Fig. 1a and b).

This limit for PFOS and PFOA was exceeded in the first 14 days of sampling for the third house, and concentrations of PFHxS were also slightly elevated, driving the higher concentrations observed in Fig. 1c. During this sampling period, the concentration of PFHxS present in surface and groundwater sources within the remit of South East Water was at least two-fold higher compared to the rest of the year.^34,54^ One possible explanation is, therefore, that the higher tap water concentrations could have been driven by a point contamination in the source water that was not effectively removed at the drinking water treatment plant. However, at no point did the concentration of individual PFAS exceed the current lowest DWI action threshold (10 ng L^−1^), and the combined PFAS concentrations did not exceed the 100 ng L^−1^ tier 3 potential hazard to health standard imposed by the DWI in 2022. Furthermore, concentrations sharply decreased over the following days, possibly due to blending water from different sources to dilute the PFAS concentrations or due to an intermittent source (SI Fig. S3).

### 3.2. Spatial assessment of PFAS concentrations in tap water from households in London

**Fig. 2:**
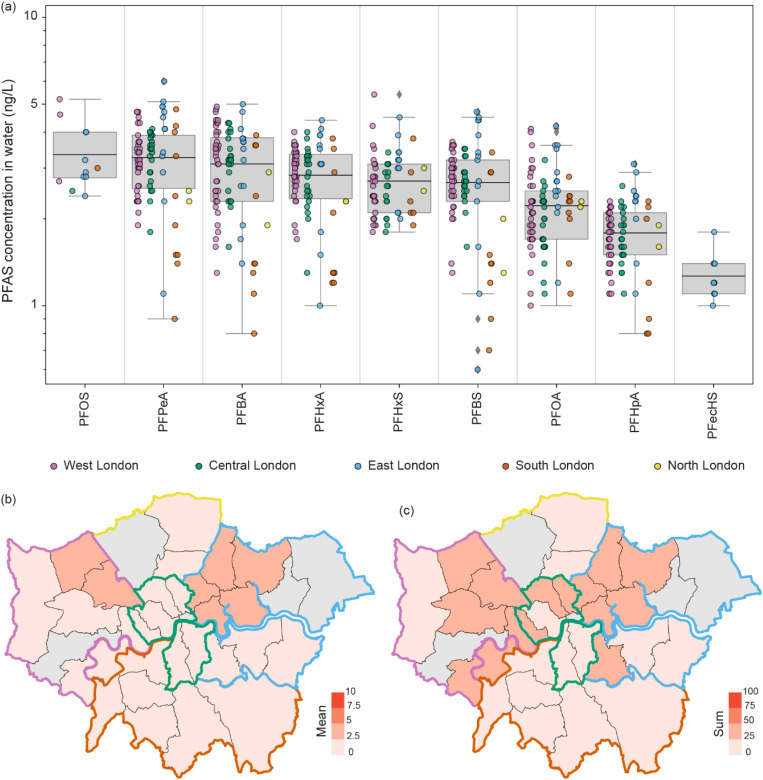
(a) Boxplot representing the range of PFAS quantified in municipal London tap water collected from taps in 89 households. The coloured dots indicate the sub-region of London where the tap water sample was collected (west = 40, central = 23, east = 14, south = 9, north = 2). Map of the Greater London boroughs with the mean individual PFAS concentration (b) and total summed PFAS concentration (c) indicated (ng L^−1^). The boundaries of the London sub-regions are coloured, and grey boroughs represent those that were not sampled. Refer to SI Table S3 for further information.

Out of the 108 sample collection kits sent to volunteer participants, 89 (82%) were successfully returned to the laboratory for analysis. Drinking water samples were collected from taps in homes from 28 of the 33 London boroughs and covered three different drinking water suppliers (Thames Water, Affinity Water and SES Water). Affinity Water abstracts drinking water from groundwater (60%) and surface waters (40%).^55^ Amongst the 3382 measurements made (representing 42% of all measurements made during this study), 11 different PFAS were detected in drinking water samples, but concentrations could only be quantified for nine compounds (PFBA, PFBS, PFecHS, PFHpA, PFHxA, PFHxS, PFOA, PFOS, and PFPeA, see SI Results S1 for more details) over the range of 0.6 ± 0.1 ng L^−1^ (PFBS) to 6.0 ± 1.5 ng L^−1^ (PFPeA, Fig. 2a). The most common PFAS compounds detected across all samples were PFBA, PFBS, PFHxA, and PFOA (all with a detection rate of 99%, SI Table S3). We found no evidence of PFAS contamination in the negative control samples collected by the participants or in the laboratory controls. Critically, no individual measured PFAS concentration was greater than the 10 ng L^−1^ limit recommended by the DWI guidelines, and the sum of all PFAS present in tap water ranged from 3 ng L^−1^ to 35 ng L^−1^, below the 100 ng L^−1^ threshold adopted by the DWI in January 2025 (Fig. 2c).^30,31^ PFOS and PFOA were quantifiable in 13% and 96% of all tap water samples, with the US EPA maximum contaminant level (MCL) of 4 ng L^−1^ exceeded in four samples (4.5% of households tested).

Across London boroughs, the highest total and average concentrations across all PFAS were observed in Waltham Forest (35 ng L; 3.7 ± 0.3 ng L^−1^), Harrow (33 ng L; 2.9 ± 2.0 ng L^−1^), and Redbridge (33 ng L; 3.6 ± 1.0 ng L^−1^) (Fig. 2b and c).

### 3.3. PFAS concentrations in public drinking water fountains in London

Twelve publicly accessible drinking water fountains were sampled on three occasions (*n* = 36, 1368 measurements). The same ten compounds as in the three-household temporal study were present above LLOQ. Six PFAS (PFBA, PFBS, PFHxA, PFHpA, PFOA, and PFPeA) were quantifiable in every sample across all sites and sampling dates. There were no significant differences (*p* > 0.05, ANOVA) between the PFAS concentrations at the fountains between the three sampling events (Fig. 1d, SI Fig. S4). All individual PFAS concentrations were well below the DWI 10 ng L^−1^ guidance value (0.9 ± 0.2 ng L^−1^ (PFecHS) to 7.7 ± 0.7 ng L^−1^ (PFHxA)), and the sum of PFAS did not exceed 100 ng L^−1^ (16 ng L^−1^ to 41 ng L^−1^, median = 25 ng L^−1^).

### 3.4. Exposure risk assessment

Assuming that an adult drinks two litres of tap water per day,^36^ the estimated daily exposure to all PFAS measured in all drinking water samples taken in this study ranged from 0.07 to 1.1 ng per kg_(bw)_ per day (mean = 0.5 ± 0.2 ng per kg_(bw)_ per day) for females, and from 0.06 to 1.0 ng per kg_(bw)_ per day (mean = 0.4 ± 0.2 ng per kg_(bw)_ per day) for males. When considering only the four PFAS compounds listed by EFSA (PFOA, PFOS, PFNA, and PFHxS), the estimated TWI from drinking water ranged from 0.2 to 3.7 ng per kg_(bw)_ per week (mean = 0.9 ± 0.7 ng per kg_(bw)_ per week) for females, and from 0.2 to 3.1 ng per kg_(bw)_ per week (mean = 0.8 ± 0.6 ng per kg_(bw)_ per week) for males. The estimated PFAS exposure is higher for females than for males due to their lower body weight.

### 3.5. Comparison with routine government agency measurements at water treatment works

In this work, across all samples collected, all PFAS measurements were below 10 ng L^−1^ both within and outside of London (Table 1). In other data sources, the proportion of measurements above 10 ng L^−1^ was found to be much higher in London than in the rest of England for the EA dataset (2.6% *vs.* 0.6%, respectively) and DWI untreated (5.1% *vs.* 0.3%) and treated (3.0% *vs.* 0.3%) water measurements for the PFAS compounds detected (Table 1). The highest proportion of measurements above 100 ng L^−1^ (Tier 3) was observed in the DWI untreated water sources for London (0.7% *vs.* 0.01% for the rest of England). However, the proportion of measurements was zero in the treated water for London and 0.002% in the treated water for the rest of England.

**Table 1:** Counts (*n*) and proportions (%) of measurements below 10 ng L^−1^, between 10 and 100 ng L^−1^ and above or equal to 100 ng L^−1^ for all PFAS compounds reported in the Drinking Water Inspectorate 2022 Chief Inspector's report,^35^ the Environment Agency 2022, 2023, and 2024 datasets,^34^ and from our own measurements. Data is presented separately for London and the rest of England. For the DWI measurements, counts and proportions are reported for both untreated and treated water, and the data represent repeated sampling for some locations. The cutoff values correspond to those used by DWI for their tiered system of actions to be taken by water suppliers

Data source	Type	Region	Total count	PFAS concentrations					
				Tier 1^b^ <10 ng L^−1^		Tier 2 10–100 ng L^−1^		Tier 3 ≥100 ng L^−1^	
				*n*	%	*n*	%	*n*	%
EA	Source water	England	209 646	208 226	99.3	1316	0.6	104	0.05
		London	17 062	16 607	97.3	435	2.6	20	0.1
DWI 2022^a^	Raw water	England	163 671	163 148	99.7	501	0.3	22	0.01
		London	11 018	10 378	94.2	567	5.1	73	0.7
	Treated water	England	103 471	100 918	99.7	254	0.3	2	0.002
		London	4754	4610	97.0	144	3.0	0	0.0
This study	Tap water	England	2166	2166	100.0	0	0.0	0	0.0
		London	5814	5814	100.0	0	0.0	0	0.0

a Matched raw and treated water data available for 12 drinking water companies; details can be found in the Drinking Water Inspectorate (DWI) 2022 Chief Inspector's report.

b Values inclusive of measurements below the limit of detection (LOD).

### 3.6. Removal of PFAS using portable filter jugs

**Fig. 3:**
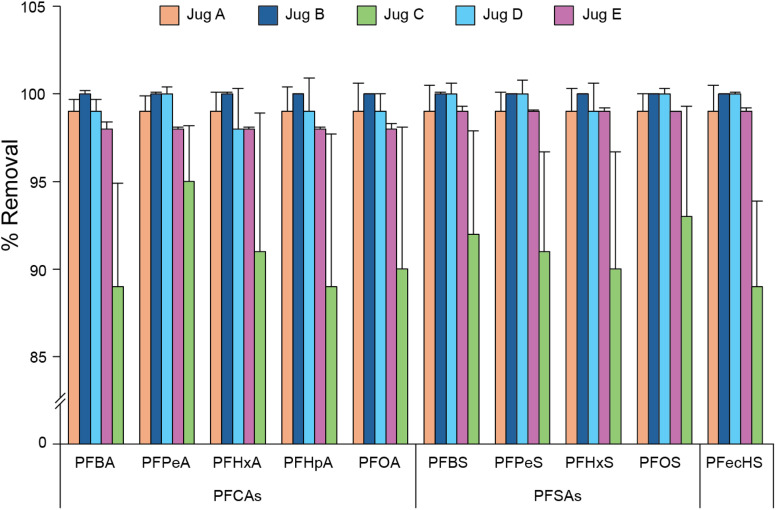
Comparison of the percentage removal of PFAS detected in drinking water across the five different commercially available filter jugs. Jug B only contains an activated carbon (AC) filter, and Jugs A, C, D, and E are advertised as containing a blend of AC and ion exchange sorbent (IEX). Error bars represent standard deviation in % removal across repeats (*n* = 3). Refer to SI Table S4 for more details.

The removal efficiency of five different filter jugs was assessed for the 38 PFAS compounds considered. Details of PFAS removal efficiencies for all jug filters and their scores are shown in SI Table S4. Across all compounds, average PFAS removal for all jugs tested was 98 ± 4% for all native PFAS compounds spiked at 50 ng L^−1^ in HPLC-grade water. According to the scoring system, the best-performing jug removed 99 ± 0.7% (score = 34) of all compounds, while the worst-performing jug had a score of 65, removing 92 ± 2% of PFAS. In relation to the PFAS detected in London tap water samples (SI Results S1), the lowest removal rate for a given compound was 89 ± 5% (PFBA, Fig. 3). Generally, PFAS removal increased for PFAS with carbon chain lengths greater than six. Between the two filter types, AC/IEX filters removed 97 ± 6% (*n* = 4 jug brands) of PFAS, with AC removing 99 ± 2% (*n* = 1 jug brand).

Critically, when using standardised HPLC-grade water spiked to individual PFAS concentrations of 50 ng L^−1^ in the laboratory, all jugs were able to reduce PFAS concentrations in water to meet the current DWI guidelines.^30^ Importantly, there was no obvious deterioration in general removal performance when filtering 100 L of tap water. All PFAS concentrations (aside from PFOA) were reduced to below the method LLOQ after the first 20 L of water filtered in this way (SI Tables S5). PFOA was briefly present above the LLOQ at the 40 L and 60 L filtrate measurements before falling back below LLOQ in subsequent measurements. The concentration of PFOA remained well below both the US EPA MCL limit and DWI guidelines at all times.

## 4 Discussion

This work, based on 210 samples and 7980 measurements from 104 locations (92 homes and 12 public water fountains), is the largest study of PFAS in household tap water in the UK. We also tested a much wider suite of PFAS compounds (*n* = 38) than other UK studies. Up to ten different PFAS were widely quantifiable in tap water samples from Greater London and nearby locations in the South of England. Importantly, all PFAS concentrations in London households were well below the DWI action threshold for individual compounds (tier 1: <10 ng L^−1^) and the cumulative sum of PFAS within an individual sample were below the 100 ng L^−1^ limit imposed by the DWI since 2022. The estimated adult daily exposure to PFAS compounds studied *via* tap water sources was determined. It is difficult to contextualise the risk posed due to the lack of guidance and research for all compounds detected. However, the estimated combined exposure to PFOA, PFOS, PFNA and PFHxS for 100% of samples was below the EFSA tolerable weekly intake of 4.4 ng per kg_(bw)_ per week.

While the EA monitors environmental waters (rivers, lakes, reservoirs and aquifers) and the DWI collates information from water companies on untreated and treated water at the point of distribution from a water treatment works, there is limited evidence of high PFAS exposure at the point of use in tap water in the UK. Testing of source, untreated, and treated water by the EA and DWI is not random, as it tends to focus on areas of potential concern and includes repeated sampling. This can lead to a higher proportion of samples being identified above 10 ng L^−1^. Water companies abstract environmental waters from multiple ground and surface water sites within their remit, with many sources blended as they enter the drinking water treatment works. In addition, water companies use various techniques (*e.g.* filtration, dilution *via* ‘blending’) to reduce concentrations of PFAS in drinking water and ensure a high water supply quality, making it difficult to determine a potential source of PFAS contamination from tap water samples alone.

Gao *et al.* (2024) assessed the concentration of ten PFAS (*i.e.*, PFOA, PFNA, PFBS, PFHxS, PFOS, FOSA, MeFOSA, EtFOSA, MeFOSE, and EtFOSE) in Birmingham and the surrounding area. Only four PFAS compounds were common between Gao *et al.*'s^56^ work and our own (PFOA, PFBS, PFHxS, and PFOS), and the average tap water concentrations in London (PFOA = 2.2 ± 0.7, PFBS = 2.7 ± 0.8, PFHxS = 2.7 ± 0.7, and PFOS = 3.3 ± 0.9 ng L^−1^) were higher than those observed in Birmingham and the surrounding cities (PFOA = 0.7 ± 0.4, PFBS = 0.8 ± 0.7, PFHxS = 0.6 ± 0.5, and PFOS = 0.7 ± 0.8 ng L^−1^). Teymoorian *et al.* assessed the PFAS concentration in a single tap water sample collected from London in 2023 as part of a global study on PFAS occurrence in tap water. Among the ∼270 PFAS assessed through targeted and suspect screening methods, eight compounds were common with our study and present at similar concentrations (PFBA = 3.9, PFBS = 3.5, PFHpA = 2.0, PFHxA = 5.1, PFHxS = 3.5, PFOA = 3.0, PFOS = 4.4, and PFPeA = 5.0 ng L^−1^).^57^

Municipal drinking water provision in England is divided between 25 different private companies. Although our study included four of these, there might be important regional differences in PFAS concentration in raw water supplies or treatment processes. Local PFAS concentrations will depend on control measures taken by the individual drinking water company, but potentially also on the characteristics of the plumbing between the treatment works and within individual households (*e.g.*, presence of PFAS in Teflon tape and other building materials^58^). Furthermore, our findings only apply to tap water supplied by municipal drinking water treatment plants. Households using a private well or spring may be subject to local contamination and should regularly monitor the quality of their drinking water. Similarly, this data does not apply to bottled water.

Exposure to PFAS *via* tap drinking water could be reduced by home jug filters, though our findings suggest this may not be needed generally in Greater London, and only in cases where PFAS contamination threshold exceedances exist. Nevertheless, PFAS removal using these filters increased with increasing carbon chain lengths greater than six within the different PFAS subgroups (*i.e.*, perfluoroalkyl carboxylic acids (PFCA) and perfluoroalkane sulfonates (PFSA)), aligning with the results of past studies.^59–61^ On average, the filter only containing AC exhibited higher removal rates compared to those that contained a blend of AC and IEX. However, only one jug model tested contained AC alone, and other tested filters that contained a blend of AC/IEX also exhibited very high PFAS removal rates (>99%). Similar to Iwabuchi and Sato,^61^ PFAS removal efficiency appeared to be more dependent on the jug model, rather than the filter sorbent. However, the exact ratio of AC to IEX and the chemical properties of the IEX sorbents in the tested filters are unknown. In addition, the physical structure of the sorbent, as well as the assembly of the filters, will likely have an influence on the PFAS removal capability. Extended use did not affect the performance of the filter jug despite the unique composition of London tap water, containing 200 to 300 ppm of CaCO_3_,^62^ which could interfere with the IEX sites by blocking absorption or displacing absorbed PFAS compounds, as noted by Zaggia *et al.* for ammonium salts.^63^ The impact of microbial growth on filter performance was not assessed in this work, though work by Mulhern *et al.* showed that PFAS removal performance was preserved over eight months for in-line AC filters.^64^

Filter jugs provide an immediate, relatively low-cost solution when: (i) PFAS concentrations in drinking water are elevated; (ii) treatment processes are insufficient to remove PFAS from raw water sources, or (iii) only locally treated drinking water is available for consumption (*e.g.*, borehole or private well). Adult daily exposure to PFAS *via* filtered tap water sources, assuming worst-case removal (85%), was slightly higher than those observed in bottled water sourced in the UK as measured by Gao *et al.* for different compounds (*F* = 0.07 ± 0.03 and *M* = 0.06 ± 0.03 ng per kg_(bw)_ per day, *n* = 10 and *F* = 0.03 and *M* = 0.02 ng per kg_(bw)_ per day, *n* = 10, respectively).^56^ Importantly, we have not tested the occurrence or removal of ultrashort-chain PFAS (<C_4_) such as trifluoroacetic acid (TFA) or trifluoromethanesulfonic acid (TFMSA) in these samples, but these have been detected in drinking water in London previously by research groups using different analytical methodologies.^65,66^

Our findings are reassuring and should alleviate possible public concerns about PFAS concentrations in drinking water in one of Western Europe's largest cities. Nevertheless, although we considered a relatively large number of PFAS in this study, there are over 4000 PFAS compounds identified, and new ones continue to be developed and manufactured.^4^ The toxicity of many of these compounds is still unclear, particularly in relation to long-term low exposures, though research is still ongoing.

## 5 Conclusion

This work is the most comprehensive study of PFAS in tap water for a major English city, encompassing 210 samples and four different drinking water companies. Up to ten different PFAS compounds were widely quantifiable in municipal drinking water samples from Greater London and two locations in the Southeast of England. The most frequently measured compounds across all samples were PFBA, PFBS, PFHxA, and PFOA. Critically, all PFAS concentrations within London were below the DWI lowest action threshold for individual compounds (10 ng L^−1^), and the cumulative sum of PFAS within an individual sample was also well below the 100 ng L^−1^ action threshold imposed by the DWI. Adult daily exposure priority PFAS compounds *via* tap water sources was determined to be below the EFSA TWI threshold for 100% of samples, though it is hard to contextualise the risk posed by these values due to the lack of guidance and research for all compounds detected. High removal rates were observed for filters that used only AC and those with a blended AC/IEX sorbent. Close monitoring of direct PFAS exposure sources, such as drinking water, and the potential health effects is critical to understanding PFAS toxicology in humans. However, our findings provide reassuring evidence about the quality of municipal drinking water in London and provide benchmark risk assessment data for future studies.

## Ethical approval

This study was conducted with ethical approval from Imperial College London (6552132).

## Author contributions

AKR: conceptualisation, methodology, resources, formal analysis, investigation, data curation, writing – original draft, writing – review & editing, project administration, funding acquisition. WHT: formal analysis, investigation, data curation, writing – review & editing. CIZO: methodology, writing – review & editing. WF: formal analysis, investigation, data curation, writing – review & editing. SD: resources, writing – review & editing. LPB: conceptualisation, methodology, writing – original draft, writing – review & editing, project administration, funding acquisition. FBP: conceptualisation, methodology, resources, writing – original draft, writing – review & editing, funding acquisition, project administration.

## Conflicts of interest

The authors declare no conflict of interest.

## Supplementary Material

VA-005-D6VA00076B-s001

## Data Availability

All data generated or analysed during this study are included in this published article and its supplementary information (SI) files. Supplementary information is available. See DOI: https://doi.org/10.1039/d6va00076b.
