# Supplementary material for: Occurrence of PFAS in municipal drinking water: a participatory case study in London, UK
Source: Env Sci Adv. 2026 May 12;5(7):1700–9. doi: 10.1039/d6va00076b (PMC13162327; doi:10.1039/d6va00076b)
Supplement: VA-005-D6VA00076B-s001 [file VA-005-D6VA00076B-s001.pdf]

Supplementary Information (SI) for Environmental Science: Advances.
This journal is © The Royal Society of Chemistry 2026

Supplementary Information

# Occurrence of PFAS in municipal drinking water: a participatory case study in London, UK

Alexandra K. Richardson[1,2,3*], Wei-Han Tien[4], Charlotte I.Z. O'Hern[2], William Francis[3], Sarah Dack[5], Leon P. Barron [2,3,4 #], and Frédéric B. Píel[1,2,3 #]

[1] *Department of Epidemiology & Biostatistics, School of Public Health, Imperial College London, London, United Kingdom.*

[2] *MRC Centre for Environment & Health, Environmental Research Group, School of Public Health, Imperial College London, London, United Kingdom.*

[3] *NIHR Health Protection Research Unit in Environmental Exposures & Health, School of Public Health, Imperial College London, London, United Kingdom.*

[4] *Department of Analytical, Environmental and Forensic Sciences, Institute of Pharmaceutical Science, King's College London, London, United Kingdom.*

[5] *UK Health Security Agency, London, United Kingdom.*

* Corresponding author: Dr Alexandra K. Richardson (a.richardson@imperial.ac.uk)

[#] Joint Senior Authorship

**Contents**

Supplementary Information ....1

Supplementary Methods ....3

Supplementary Methods S1: Sample collection....3

Supplementary Methods S2: Sample preparation ....3

Supplementary Methods S3: Instrumental Analysis ....4

Supplementary Methods S4: Testing of water filter jugs....4

Supplementary Results ....5

Supplementary Results S1: Detected PFAS....5

Supplementary Figures ....6

Supplementary Figure S1. Map of all sampling sites used in this study in England and London. ....6

Supplementary Figure S2. Contents of home sampling kits that were sent to participants. ....7

Supplementary Figure S3. Daily cumulative concentrations of PFAS in tap water. The successive daily cumulative PFAS concentration in tap water from each household, (a) to (c). The red line indicates the 100 ng/L cumulative limit for PFAS in drinking water. ....8

Supplementary Figure S4. Average PFAS concentrations at each public water fountain site over the three-sample collection timepoints. Light grey = 7$^{th}$ of March 2022, grey = 22$^{nd}$ of March 2022, dark grey = 7$^{th}$ of April 2022. ....9

Supplementary Tables....11

Supplementary Table S1. List of PFAS analytical standards, Chemical Abstract Service (CAS) number, retention time ($t_R$), and MRM transitions used for targeted analysis per compound. ....11

Supplementary Table S2. Mean ± standard deviation and median of the maximum PFAS concentrations (ng/L) reported in the literature from municipal drinking water samples taken directly from taps. ....14

Supplementary Table S3. Summary statistics for the nine PFAS quantified in tap water (ng/L) for each borough within Greater London. ....17

Supplementary Table S4. Average removal efficiencies (n = 3) ± standard deviation of PFAS compounds for all filter jugs tested. The PFAS removal score is indicated in parenthesis. ....21

Supplementary Table S5. Average PFAS concentration in London tap water and average concentration in tap water after filtration. ....22

Supplementary Table S6. Comparison between calibration lines and the quantification results of QCs (average ± standard deviation) prepared using the Andrew+ robot and the laboratory user for all PFAS compounds. The mean absolute error (MAE) between the slope, intercept and QC quantification values is presented. ....23

Supplementary Table S7. Method performance characteristics for samples prepared in laboratory tap water, analysed via direct injection. LOD: limit of detection; LLOQ: Lower limit of quantification. ....25

Supplementary Table S8. Method performance characteristics for samples prepared in laboratory HPLC-grade water, analysed via direct injection. ....26

Supplementary Table S9. Method performance characteristics for samples prepared in HPLC-grade water using the evaporation concentration method. ....27

Supplementary References ....28

## Supplementary Methods

### Supplementary Methods S1: Sample collection

The sampling kits were developed with input and guidance from members of the Public and Community Oversight Group of the MRC Centre for Environment & Health, and community representatives of the NERC West London Health Home and Environment Study.

Each kit contained a detailed set of instructions and all equipment necessary for sample collection (three 30 mL Nalgene bottles (labelled A, C1 (pre-filled with HPLC-grade bottled water) and C2), two plastic bags and return postage supplies, **Supplementary Figure S2**). Participants were instructed to run the tap for at least 60 seconds before sample collection to ensure fresh water was being pulled from the supply before rinsing bottle A three times and then collecting the final sample. Participants were then required to transfer the contents of bottle C1 to C2, which acted as a field blank for the sample collection. All bottles were then double-bagged and returned to the laboratory via the local postal service.

### Supplementary Methods S2: Sample preparation

To avoid contamination often introduced through the sample preparation process, a direct injection protocol was used for the analysis of all samples. In summary, 900 µL of sample water (collected tap water and field blanks) were spiked with 100 µL of MeOH containing IS (to a final concentration of 25 ng $L^{-1}$). All samples were prepared and analysed in certified polypropylene PFAS-free HPLC vials with a polypropylene septum. Standards and methanol aliquots were added to the water samples using the Andrew+ robot system (Waters, Wilmslow, UK). Refer to **Supplementary Methods S3** for instrumental analysis parameters. Refer to **Supplementary Table S6** for a performance evaluation of the Andrew+.

For quantification purposes, an external calibration curve was prepared in HPLC-grade bottled water over the range of 0.5 ng $L^{-1}$ to 100 ng $L^{-1}$ as above, maintaining a SIL-IS concentration of 25 ng $L^{-1}$ across all samples. Where possible, quantification was performed using the peak area ratio between the native PFAS compounds and corresponding SIL-IS. Quality control (QC) samples were prepared as above, spiked to 6, 18, and 60 ng/L with native PFAS compounds. Controls consisting of only MeOH and HPLC-grade bottled water (900 µL of each reagent, no additional SIL-IS added) were analysed to account for contamination from those reagents.

All test samples, field blanks, the 18 ng $L^{-1}$ QC, and controls were randomised within the batch file with the total run time less than 48 hours per batch. The standard curve was evenly spaced throughout the batch file with the 6 and 60 ng $L^{-1}$ QCs bracketing the whole run, in-line with the recommendations from the US EPA 533 method for PFAS analysis [1]. Refer to **Supplementary Table S7** and **Supplementary Table S8** for details of method performance assessment.

**Supplementary Methods S3: Instrumental Analysis**

All reagents used were at least HPLC-MS grade or higher unless stated otherwise and confirmed to be PFAS-free by in-house testing. Methanol (MeOH) and bottled water was obtained from VWR Scientific (Leicestershire, UK) and Fisher Scientific (Leicestershire, UK), respectively. For quantitative targeted analysis, a standard mix of 64 PFAS standards, including 19 stable isotopic-labelled internal standards (SIL-IS) with a purity of ≥ 97% was used. Refer to Table S1 for full details.

All water samples were analysed using a Shimadzu Nexera X2 LC and LCMS-8060 (Shimadzu Corporation, Kyoto, Japan) instrument with a PFAS delay column (50 x 2.1 mm, Thames Restek, High Wycombe, UK) fitted before the autosampler. Separations were performed over nine and a half minutes using a Force C18 column (50 x 2.1 mm, 3 um, Thames Restek, High Wycombe, UK) fitted with a complimentary guard column (Force C18, 5.0 x 2.1 mm, 5 um, Thames Restek, High Wycombe, UK) with 10 mM ammonium acetate (aq) as mobile phase A (MPA) and MeOH as mobile phase B (MPB). Both the delay and analytical columns were held at 40 ºC throughout the analysis, the flow rate was 0.5 mL $min^{-1}$ and 50 mL of sample was injected onto the column. The gradient program consisted of an initial hold at 30% MPB for 0.51 min, then a linear ramp to 60% at 1.5 min, then a second ramp to 90% at 5 min, then an increase to 95% at 6 min, followed by a hold at 95% MPB for 1 min, the system was re-equilibrated at starting conditions for an additional 2 min.

During the multiple reaction monitoring, the interface temperature was maintained at 300°C, the desolvation line (DL) temperature at 150°C, and the heat block at 200°C. The gas flows were set at 3, 5, and 15 L $min^{-1}$ for the nebulizing, drying, and heating gas, respectively.

**Supplementary Methods S4: Testing of water filter jugs**

All jugs were washed and the filters were conditioned with HPLC-grade water before use as per their respective instruction manuals. The removal of PFAS was assessed via recovery by spiking 500 mL of bottled HPLC-grade water to 50 ng/L for all native PFAS compounds, which was allowed to fully pass through the filter before analysis.

Unspiked bottled HPLC-grade water was used as a control. In addition, the best performing filter for PFAS removal, was subjected to a "real world use" scenario, whereby 100 L of tap water from two different sources (Site A and Site B) within London was passed through the filter (a new conditioned filter was used at each location) with samples collected every 20 L from the filtrate.

Filtrate samples were prepared for analysis by fully evaporating 5 mL of water at 40 ºC under a gentle stream of nitrogen. Samples were then reconstituted in 200 µL of starting mobile phase conditions (70:30 H2O:MeOH) and transferred to certified polypropylene PFAS-free HPLC insert vials. Refer to **Supplementary Table S9** for details of method performance assessment.

## Supplementary Results

### Supplementary Results S1: Detected PFAS

Across the whole study, 11 different PFAS compounds (PFBA, PFBS, PFecHS, PFHpA, PFHxA, PFHxS, PFNA, PFOA, PFOS, PFPeA, PFPeS) were present above the method's limit of detection (LOD). The limit of detection is defined as the lowest analyte concentration that can be detected by the analytical method with confidence [2]. To assign a reliable value to the concentration of PFAS in a sample, the concentration must first exceed the method's lower limit of quantification (LLOQ). This is the lowest value at which the instrument performance is acceptable for this application [2]. Refer to **Supplementary Table S7** and **Supplementary Table S8** for the LOD and LLOQ values used for each PFAS compound in this work.

## Supplementary Figures

**Supplementary Figure S1. Map of all sampling sites used in this study in England and London.**

Grey shaded regions indicate counties where tap water samples were collected across the whole study. Areas with a red border contain the households used to assess daily variability. Dotted regions contain the public water drinking fountains that were sampled.

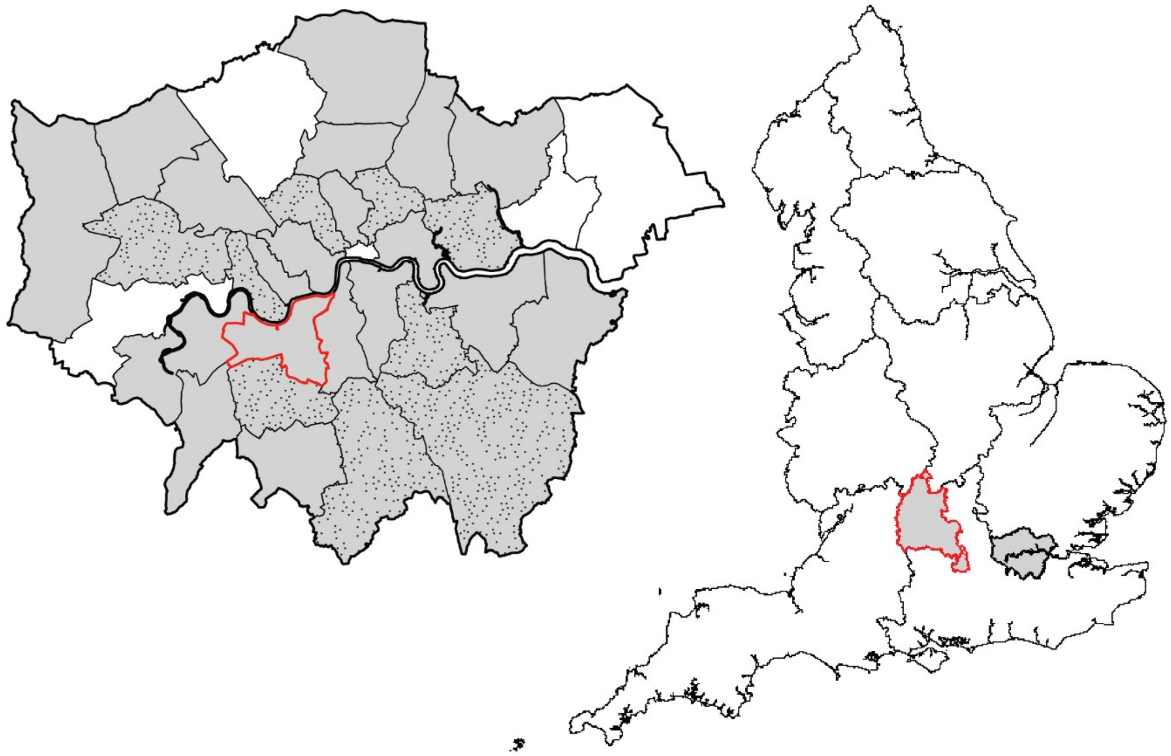

**Supplementary Figure S2. Contents of home sampling kits that were sent to participants.**

(a) Letter-sized cardboard box that contained all components, (b) plastic bags for double bagging water samples, (c) pre-labelled return envelope, (d) 30 mL polypropylene Nalgene bottles for sample collection (A) and field blanks (C1 and C2), (e) permanent marker for labelling, (f) sample collection instructions, and (g) box closure stickers.

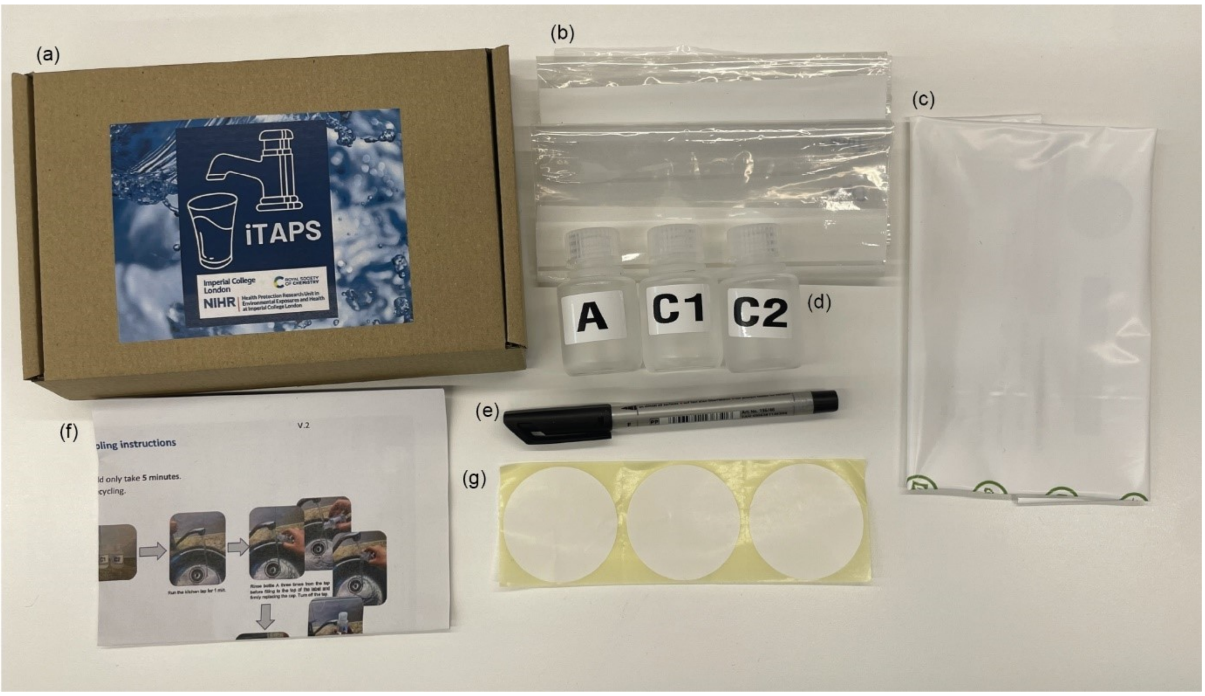

**Supplementary Figure S3. Daily cumulative concentrations of PFAS in tap water.** The successive daily cumulative PFAS concentration in tap water from each household, (a) to (c). The red line indicates the 100 ng/L cumulative limit for PFAS in drinking water.

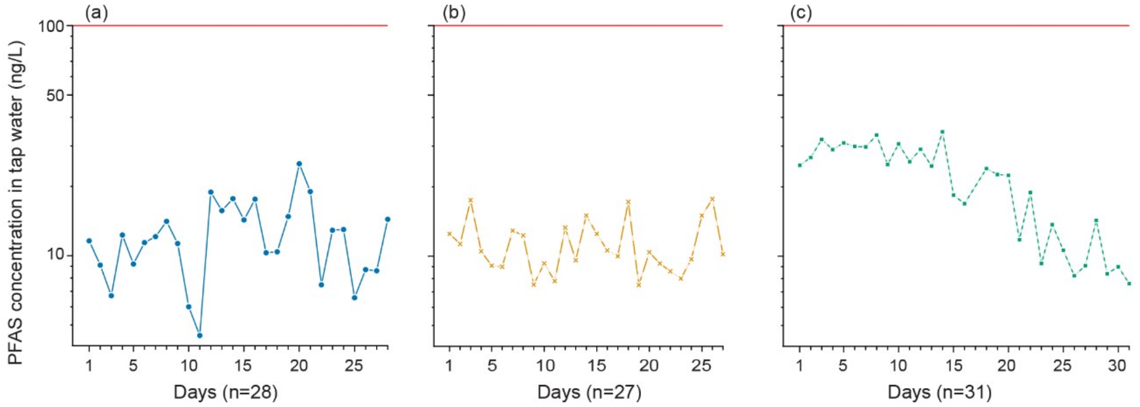

**Supplementary Figure S4. Average PFAS concentrations at each public water fountain site over the three-sample collection timepoints.** Light grey = 7th of March 2022, grey = 22nd of March 2022, dark grey = 7th of April 2022.

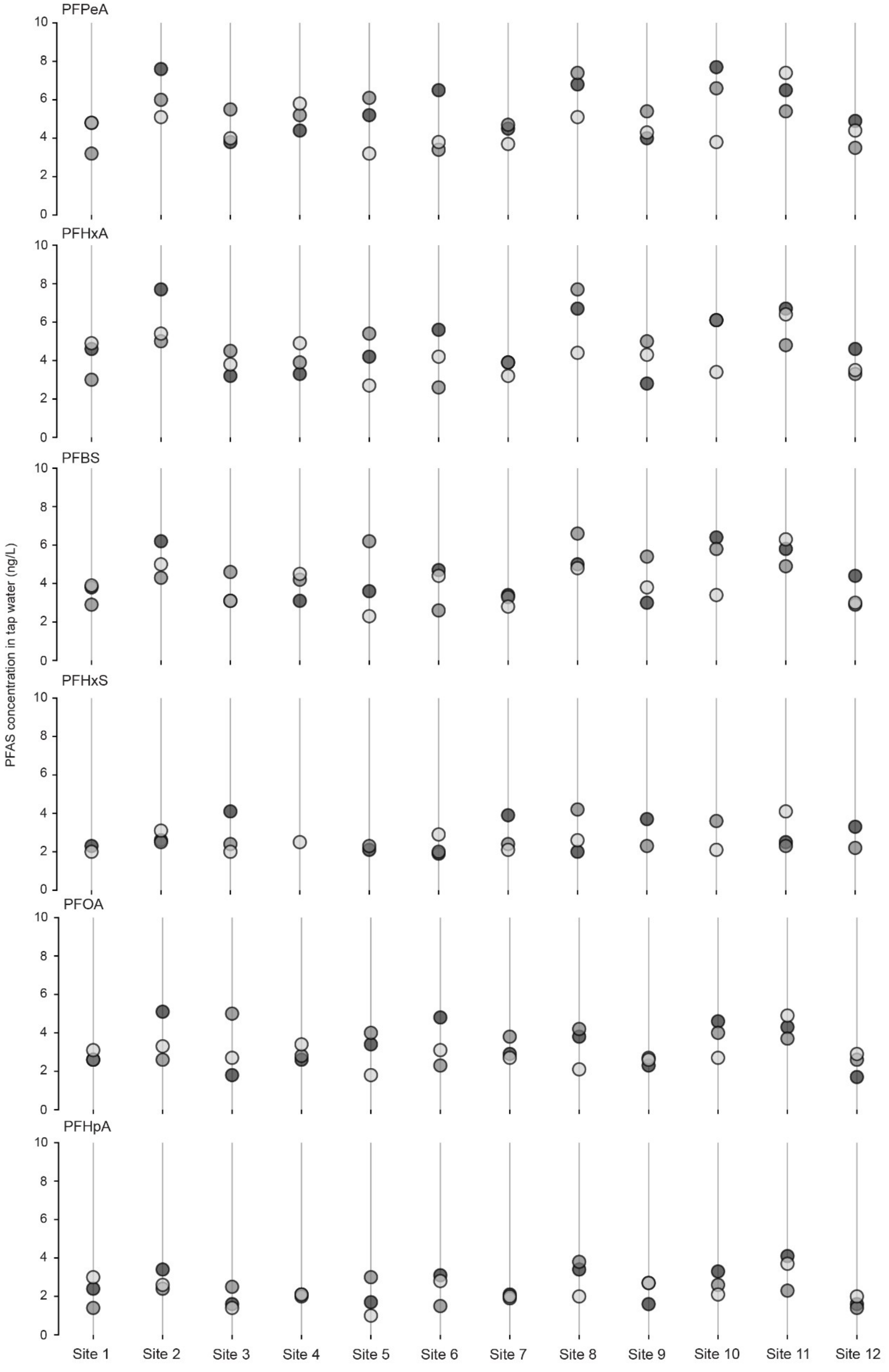

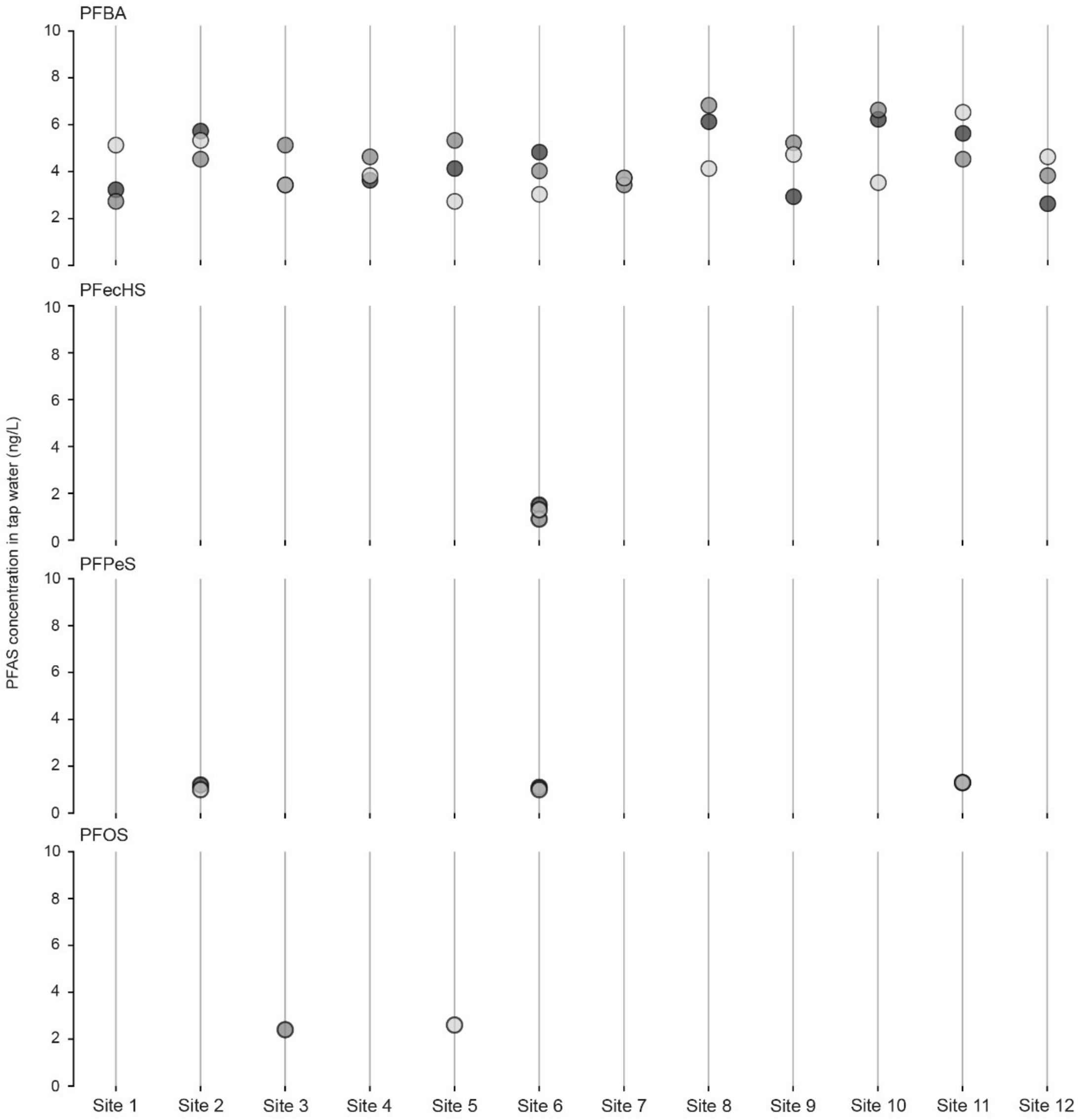
PFBA
PFecHS
PFPeS
PFOS
PFAS concentration in tap water (ng/L)
0
2
4
6
8
10
Site 1
Site 2
Site 3
Site 4
Site 5
Site 6
Site 7
Site 8
Site 9
Site 10
Site 11
Site 12

## Supplementary Tables

**Supplementary Table S1.** List of PFAS analytical standards, Chemical Abstract Service (CAS) number, retention time ($t_R$), and MRM transitions used for targeted analysis per compound.

| # | Abbreviation | Name | CAS | MRM transition | $t_R$ (min) |
|---|---|---|---|---|---|
| *Native standards* | | | | | |
| 1 | 4:2 FTS | Sodium 1H,1H,2H,2H-perfluorohexane sulfonate | 3871-99-6 | 326.75 > 307.05*<br>326.75 > 81.05 | 2.46 |
| 2 | 5:3 FTCA | 2H,2H,3H,3H-Perfluorooctanoic Acid | 914637-49-3 | 341.00 > 217.20*<br>341.00 > 237.20 | 2.94 |
| 3 | 6:2 Cl-PFESA | 9-Chlorohexadecafluoro-3-Oxanone-1-Sulfonic Acid | 73606-19-6 | 531.05 > 351.15*<br>531.05 > 34.90 | 3.88 |
| 4 | 6:2 FTS | Sodium 1H,1H,2H,2H-perfluorooctane sulfonate | 375-95-1 | 426.75 > 407.00*<br>426.75 > 81.10 | 3.24 |
| 5 | 7:3 FTCA | 2H,2H,3H,3H-Perfluorodecanoic Acid | 72629-94-8 | 440.90 > 317.15*<br>440.90 > 337.20 | 3.79 |
| 6 | 8:2 Cl-PFESA | 11-chloroeicosafluoro-3-oxaundecane-1-sulfonic acid | 83329-89-9 | 631.10 > 450.95* | 4.56 |
| 7 | 8:2 FTS | Sodium 1H,1H,2H,2H-perfluorodecane sulfonate | 98789-57-2 | 527.05 > 506.95*<br>527.05 > 81.00 | 4.06 |
| 8 | DONA | 4,8-dioxa-3H-Perfluorononanoic Acid | 919005-14-4 | 376.95 > 251.15*<br>376.95 > 85.10 | 2.90 |
| 9 | HFPO-TA | Hexafluoropropylene oxide trimer acid | 13252-14-7 | 495.55 > 398.15* | 3.50 |
| 10 | L-PFDoS | Sodium perfluoro-1-dodecanesulfonate | 2795-39-3 | 699.00 > 80.05*<br>699.00 > 99.00 | 4.99 |
| 11 | L-PFUdS | Sodium perfluoro-1-undecanesulfonate | 376-06-7 | 649.00 > 80.00*<br>649.00 > 99.05 | 4.72 |
| 12 | MeFOSA | N-Methyl-perfluorooctanesulfonamide | 16517-11-6 | 511.85 > 219.20*<br>511.85 > 269.05 | 5.03 |
| 13 | N-EtFOSAA | 2-(N-ethylperfluorooctanesulfonamido) acetic acid | 375-22-4 | 583.70 > 419.00*<br>583.70 > 526.00 | 4.42 |
| 14 | N-MeFOSAA | 2-(N-methylperfluorooctanesulfonamido) acetic acid | 29420-49-3 | 570.05 > 418.95*<br>570.05 > 482.95 | 4.24 |
| 15 | PFBA | Perfluorobutanoic acid | 27619-93-8 | 212.80 > 169.15* | 1.19 |
| 16 | PFBS | Perfluorobutyl sulfonate | 335-76-2 | 298.75 > 80.10*<br>298.75 > 99.00 | 2.21 |
| 17 | PFDA | Perfluorodecanoic acid | 1260224-54-1 | 512.65 > 468.95*<br>512.65 > 219.05 | 4.06 |
| 18 | PFDoA | Perfluorododecanoic acid | 441296-91-9 | 612.65 > 569.00*<br>612.65 > 169.15 | 4.74 |
| 19 | PFDS | Perfluorodecane sulfonate | 67905-19-5 | 598.80 > 80.10*<br>598.80 > 99.10 | 4.40 |

| | | | | | |
|---|---|---|---|---|---|
| 20 | PFecHS | Perfluoroethylcyclohexane sulphonate | 335-24-0 | 460.80 > 381.10*<br>460.80 > 99.05 | 3.20 |
| 21 | PFEESA | Perfluoro(2-ethoxyethane)sulphonic acid | 113507-82-7 | 314.90 > 135.15* | 2.35 |
| 22 | PFHpA | Perfluoroheptanoic acid | 27619-96-1 | 362.75 > 319.05*<br>362.75 > 169.15 | 2.85 |
| 23 | PFHpS | Perfluoroheptane sulfonate | 375-85-9 | 448.85 > 80.15*<br>448.85 > 99.00 | 3.27 |
| 24 | PFHxA | Perfluorohexanoic acid | 812-70-4 | 312.95 > 269.05*<br>312.95 > 119.10 | 2.50 |
| 25 | PFHxDA | Perfluoro-n-hexadecanoic acid | 2706-90-3 | 813.00 > 768.85*<br>813.00 > 169.20 | 5.67 |
| 26 | PFHxS[a] | Perfluorohexane sulfonate | 2806-15-7 | 398.85 > 99.05*<br>398.85 > 80.00 | 2.87 |
| 27 | PFMOBA | Perfluoro-4-methoxybutanic acid | 863090-89-5 | 278.85 > 85.05* | 2.25 |
| 28 | PFMOPrA | Perfluoro-3-methoxypropanoic acid | 377-73-1 | 228.95 > 85.10* | 1.66 |
| 29 | PFNA | Perfluorononanoic acid | 13252-13-6 | 463.05 > 419.00*<br>463.05 > 219.25 | 3.67 |
| 30 | PFNS | Perfluorononane sulfonate | 21934-50-9 | 548.85 > 80.05*<br>548.85 > 99.10 | 4.05 |
| 31 | PFOA | Perfluorooctanoic acid | 754-91-6 | 412.85 > 369.10*<br>412.85 > 169.15 | 3.26 |
| 32 | PFODA | Perfluoro-n-octadecanoic acid | 630402-22-1 | 913.00 > 869.00*<br>913.00 > 169.20 | 5.98 |
| 33 | PFOS[b] | Perfluorooctyl sulfonate | 307-24-4 | 498.95 > 80.00*<br>498.65 > 99.00 | 3.67 |
| 34 | PFPeA | Perfluoropentanoic acid | 27619-94-9 | 263.00 > 219.25*<br>263.00 > 69.10 | 2.14 |
| 35 | PFPeS | Perfluoropentane sulfonate | 307-55-1 | 349.05 > 80.10*<br>349.05 > 99.00 | 2.53 |
| 36 | PFTeDA | Perfluorotetradecanoic acid | 151772-58-6 | 712.65 > 668.95*<br>712.65 > 169.20 | 5.26 |
| 37 | PFTrDA | Perfluorotridecanoic acid | 2991-50-6 | 662.85 > 619.00*<br>662.85 > 169.10 | 5.01 |
| 38 | PFUdA | Perfluoroundecanoic acid | 24448-09-7 | 562.65 > 519.15*<br>562.65 > 269.20 | 4.42 |
| *Internal standards* | | | | | |
| 1 | d3-N-MeFOSAA | d3-N-methyl perfluorooctanesulfonamidoacetic acid | - | 572.50 > 419.10*<br>572.50 > 515.15 | 4.24 |
| 2 | d5-N-EtFOSAA | d5-N-ethyl perfluorooctane sulfonamido acetic acid | - | 588.95 > 419.00*<br>588.95 > 531.15 | 4.41 |

| | | | | | |
|---|---|---|---|---|---|
| 3 | M2-4:2 FTS | M2-Sodium 1H,1H,2H,2H-perfluorohexane sulfonate | - | 328.90 > 308.95*<br>328.90 > 80.95 | 2.47 |
| 4 | M2-6:2 FTS | M2-Sodium 1H,1H,2H,2H-perfluorooctane sulfonate | - | 428.50 > 409.00*<br>428.50 > 81.05 | 3.24 |
| 5 | M2-8:2 FTS | M2-Sodium 1H,1H,2H,2H-perfluorodecane sulfonate | - | 528.50 > 509.10*<br>528.50 > 81.15 | 4.06 |
| 6 | M2-PFHxDA | M2-Perfluoro-n-hexadecanoic acid | - | 815.00 > 770.00*<br>815.00 > 169.05 | 5.66 |
| 7 | M2-PFTeDA | M2-Perfluorotetradecanoic acid | - | 714.90 > 670.10*<br>714.90 > 169.10 | 5.26 |
| 8 | M3-PFBS | M3-Perfluorobutyl sulfonate | - | 301.50 > 80.00*<br>301.50 > 99.05 | 2.22 |
| 9 | M3-PFHxS | M3-Perfluorohexane sulfonate | - | 401.50 > 80.05*<br>401.50 > 98.95 | 2.87 |
| 10 | M4-PFHpA | M4-Perfluoroheptanoic acid | - | 366.90 > 322.10*<br>366.90 > 172.10 | 2.85 |
| 11 | M6-PFDA | M6-Perfluorodecanoic acid | - | 518.50 > 474.05* | 4.06 |
| 12 | M7-PFUdA | M7-Perfluoroundecanoic acid | - | 569.90 > 525.00*<br>569.90 > 274.00 | 4.42 |
| 13 | M8-PFOA | M8-Perfluorooctanoic acid | - | 420.50 > 376.05*<br>420.50 > 172.15 | 3.25 |
| 14 | M8-PFOS | M8-Perfluorooctyl sulfonate | - | 506.50 > 80.00*<br>506.50 > 98.95 | 3.67 |
| 15 | M9-PFNA | M9-Perfluorononanoic acid | - | 472.00 > 427.15*<br>472.00 > 223.1 | 3.66 |
| 16 | M-PFDoA | M-Perfluorododecanoic acid | - | 614.50 > 570.05*<br>614.50 > 169.20 | 4.74 |

* Quantifier ion

[a] Linear and branched isomers are present in the mix. Purity of linear form = 81 %

[b] Linear and branched isomers are present in the mix. Purity of linear form = 79 %

**Supplementary Table S2.** Mean ± standard deviation and median of the maximum PFAS concentrations (ng/L) reported in the literature from municipal drinking water samples taken directly from taps.

| Country | | PFOS | PFOA | PFNA | PFHxS | Others‡ | References |
|---|---|---|---|---|---|---|---|
| Australia | Mean ± SD | 1.5 ± 4 | 1.0 ± 3 | 0.03 ± 0.006 | 8.8 ± 6 | 0.4 ± 1 | |
| | Median | 0.04 | 0.1 | 0.03 | 8.8 | 0.03 | 3–5 |
| | Maximum | 15.6 | 9.7 | 0.03 | 14..4 | 6.8 | |
| Belgium | Mean ± SD | 1.5 ± 1 | - | 0.3 ± 0.07 | 0.7 ± 0.3 | 3.3 ± 3 | |
| | Median | 1.5 | - | 0.3 | 0.7 | 2.9 | 6–8 |
| | Maximum | 2.7 | 2.7 | 0.3 | 0.9 | 12.0 | |
| Brazil | Mean ± SD | - | - | - | - | 22.2 ± 14 | |
| | Median | - | - | - | - | 18 | 9 |
| | Maximum | 44 | 46 | 47 | - | 42 | |
| Burkina Faso | Mean ± SD | - | - | - | - | 5.9 ± 14 | |
| | Median | - | - | - | - | 0.9 | 10 |
| | Maximum | 3.9 | 1.9 | 0.4 | 1 | 39.3 | |
| Canada | Mean ± SD | 10.4 ± 7 | 41.3 ± 63 | 1.8 ± 1 | 25 ± 18 | 3.3 ± 12 | |
| | Median | 9 | 6.5 | 1.1 | 31.0 | 0.2 | 10–13 |
| | Maximum | 20.2 | 150.1 | 3.8 | 43.2 | 104.6 | |
| China | Mean ± SD | 4.7 ± 5 | 31.3 ± 36 | 1.7 ± 2 | 18.3 ± 36 | 12.8 ± 29 | |
| | Median | 2.3 | 15.5 | 1.1 | 5.1 | 1.9 | 10,14–27 |
| | Maximum | 14.8 | 115.4 | 7.0 | 139.7 | 175.3 | |
| Czechia | Mean ± SD | 8.8 ± 3 | 65.4 ± 43 | 6.9 ± 6 | 1.3 ± 0.5 | 12.6 ± 24 | |
| | Median | 8.8 | 65.4 | 6.9 | 1.3 | 0.5 | 28,29 |
| | Maximum | 11.8 | 108.0 | 13.3 | 1.8 | 97.7 | |
| England | Mean ± SD | 3.3 ± 1 | 2.4 ± 0.6 | 0.5 ± 0.3 | 2.3 ± 1 | 0.8 ± 1 | |
| | Median | 3.3 | 2.4 | 0.5 | 2.3 | 0.1 | 13,19 |
| | Maximum | 4.4 | 2.9 | 0.8 | 3.5 | 5.1 | |
| France | Mean ± SD | 5.6 ± 4 | 17.5 ± 10 | 2.2 ± 1 | 7.2 ± 5 | 3.1 ± 10 | |
| | Median | 4.4 | 19.8 | 2.4 | 7.3 | 0.2 | 6,9,13,30 |
| | Maximum | 12.6 | 29.0 | 3.8 | 12.8 | 72.2 | |
| Germany | Mean ± SD | 6.3 ± 8 | 88.5 ± 193 | 0.9 ± 0.6 | 9.5 ± 3 | 8.3 ± 14 | |
| | Median | 2.8 | 2.5 | 0.9 | 9.5 | 4.4 | 6,8,31–34 |
| | Maximum | 22.0 | 519.0 | 1.4 | 12.1 | 77.0 | |
| Ghana | Mean ± SD | - | - | - | - | 0.1 ± 0.01 | |
| | Median | - | - | - | - | 0.1 | 35 |
| | Maximum | 168 | 190 | - | - | 0.1 | |
| Greece | Mean ± SD | - | 1.9 ± 2 | - | - | 0.4 ± 1 | |
| | Median | - | 1.9 | - | - | 0.1 | 13,36 |
| | Maximum | 0.4 | 3.6 | 0.04 | 0.9 | 15.1 | |
| Hong Kong | Mean ± SD | - | - | - | - | 2.8 ± 3 | |
| | Median | - | - | - | - | 1.5 | 37 |
| | Maximum | 8.6 | 39.7 | 1 | - | 7.6 | |
| India | Mean ± SD | - | - | - | - | - | |
| | Median | - | - | - | - | - | 11 |
| | Maximum | 8.4 | 2.0 | - | - | - | |

| | | | | | | | |
|---|---|---|---|---|---|---|---|
| Iran | Mean ± SD | - | - | - | - | - | 38 |
| | Median | - | - | - | - | - | |
| | Maximum | 99.2 | 105.6 | - | - | - | |
| Ireland | Mean ± SD | - | - | - | - | - | 39 |
| | Median | - | - | - | - | - | |
| | Maximum | 0.8 | 1.8 | 0.4 | - | 15.1 | |
| Italy | Mean ± SD | 2.9 ± 3 | 3.5 ± 2 | 0.3 ± 0.2 | 0.8 ± 0.5 | 0.7 ± 1 | 6,8,13 |
| | Median | 1.5 | 4.9 | 0.2 | 1.1 | 0.1 | |
| | Maximum | 6.9 | 5.1 | 0.5 | 1.2 | 4.5 | |
| Ivory Coast | Mean ± SD | - | - | - | - | 0.5 ± 0.3 | 10 |
| | Median | - | - | - | - | 0.4 | |
| | Maximum | 1.3 | 0.3 | - | 0.8 | 0.9 | |
| Japan | Mean ± SD | 1.4 ± 0.2 | 11 ± 7 | - | - | 2 ± 2 | 10,11 |
| | Median | 1.4 | 11 | - | - | 1.9 | |
| | Maximum | 1.6 | 18.0 | 4.5 | 0.7 | 4.6 | |
| Malaysia | Mean ± SD | - | - | - | - | 9.7 ± 6 | 40 |
| | Median | - | - | - | - | 9.1 | |
| | Maximum | 19 | 171.3 | 48.5 | 1.4 | 17.2 | |
| Mexico | Mean ± SD | - | - | - | - | 1.2 ± 3 | 6,13 |
| | Median | - | - | - | - | 0.1 | |
| | Maximum | 1.4 | 1.5 | 0.3 | 2.1 | 11.7 | |
| Netherlands | Mean ± SD | 2.8 ± 2 | 8.9 ± 2 | - | 1.6 ± 0.5 | 7.5 ± 6 | 36,41 |
| | Median | 2.6 | 8.6 | - | 1.3 | 5.2 | |
| | Maximum | 5.0 | 11.1 | - | 2.3 | 19.8 | |
| Norway | Mean ± SD | 0.4 ± 0.2 | 1.8 ± 0.4 | - | - | 0.5 ± 0.2 | 8,42 |
| | Median | 0.4 | 1.8 | - | - | 0.4 | |
| | Maximum | 0.6 | 2.2 | - | 0.1 | 0.8 | |
| Portugal | Mean ± SD | - | - | - | - | 1.3 ± 1 | 6 |
| | Median | - | - | - | - | 0.6 | |
| | Maximum | 0.3 | 0.4 | - | 0.2 | 3.2 | |
| Singapore | Mean ± SD | - | - | - | - | 2 ± 2 | 43 |
| | Median | - | - | - | - | 1.5 | |
| | Maximum | 2.2 | 1.8 | 1.3 | 1.0 | 4.6 | |
| South Korea | Mean ± SD | 2.4 ± 0.9 | 15 ± 10 | 2.5 ± 1 | 64.6 ± 88 | 17.7 ± 54 | 44–46 |
| | Median | 1.8 | 14 | 3 | 2.4 | 1.3 | |
| | Maximum | 3.6 | 27.7 | 3.6 | 189.6 | 224.0 | |
| Spain | Mean ± SD | 62 ± 93 | 13.1 ± 14 | 17.2 ± 16 | 10.6 ± 12 | 9.1 ± 12 | 6,9,13,32,47–49 |
| | Median | 6.2 | 6.6 | 9.6 | 3.0 | 3.2 | |
| | Maximum | 258.0 | 35.0 | 46.0 | 28.0 | 58.0 | |
| Sweden | Mean ± SD | - | - | - | - | 1.4 ± 1 | 8 |
| | Median | - | - | - | - | 1 | |
| | Maximum | 8.8 | 6.2 | 0.4 | 2.5 | 2.9 | |
| Thailand | Mean ± SD | 37.9 ± 16 | - | - | - | - | 50 |
| | Median | 37.9 | - | - | - | - | |
| | Maximum | 54.2 | - | - | - | - | |

| | | | | | | | |
|---|---|---|---|---|---|---|---|
| Turkey | Mean ± SD | - | - | - | - | 2.9 ± 4 | 51 |
| | Median | - | - | - | - | 1.7 | |
| | Maximum | 2.0 | 2.4 | 0.4 | 2.2 | 11.3 | |
| United States of America | Mean ± SD | 27.2 ± 33 | 22.3 ± 29 | 2.4 ± 2 | 10.7 ± 11 | 18.3 ± 45 | 6,10,11,13,52–61 |
| | Median | 14.5 | 8.3 | 2.3 | 4.7 | 0.8 | |
| | Maximum | 98.6 | 108.0 | 7.9 | 34.3 | 295.0 | |
| Vietnam | Mean ± SD | - | - | - | - | 1 ± 0.2 | 62 |
| | Median | - | - | - | - | 1 | |
| | Maximum | - | 0.5 | 0.2 | 0.1 | 1.2 | |

‡ 268 unique PFAS compounds reported across all studies; refer to individual references for specific compounds.

**Supplementary Table S3.** Summary statistics for the nine PFAS quantified in tap water (ng/L) for each borough within Greater London.

| | | PFBA | PFBS | PFecHS | PFHpA | PFHxA | PFHxS | PFOA | PFOS | PFPeA |
|---|---|---|---|---|---|---|---|---|---|---|
| Greater London | | | | | | | | | | |
| | DR% (> LOD) | 99 | 99 | 49 | 98 | 99 | 94 | 99 | 82 | 98 |
| | DR% (> LLOQ) | 99 | 99 | 10 | 94 | 98 | 72 | 96 | 13 | 98 |
| | Mean ± SD | 3.1 ± 1.0 | 2.7 ± 0.8 | 1.3 ± 0.3 | 1.8 ± 0.5 | 1.8 ± 0.5 | 2.7 ± 0.7 | 2.2 ± 0.7 | 3.3 ± 0.9 | 3.3 ± 1.0 |
| | Median | 3.2 | 2.7 | 1.2 | 1.8 | 3.0 | 2.6 | 2.2 | 3.0 | 3.3 |
| | Range | < LOD – 5.0 | < LOD – 4.7 | < LOD – 1.8 | < LOD – 3.1 | < LOD – 4.4 | < LOD – 5.4 | < LOD – 4.2 | < LOD – 5.2 | < LOD – 6.0 |
| West London | | | | | | | | | | |
| Brent | DR% (> LLOQ) | 100 | 100 | - | 100 | 100 | 100 | 100 | 33 | 100 |
| (3) | Mean ± SD | 3.6 ± 1.6 | 2.8 ± 0.7 | < LLOQ | 1.7 ± 0.5 | 3.0 ± 0.6 | 3.1 ± 1.0 | 3.1 ± 0.8 | 4.6 ± 0.2 | 3.5 ± 1.3 |
| | Median | 4.0 | 2.7 | - | 1.9 | 3.0 | 2.7 | 3.0 | - | 3.7 |
| | Range | 1.8 – 4.9 | 2.1 – 3.6 | - | 1.1 – 2.1 | 2.3 – 3.6 | 2.4 – 4.2 | 2.3 – 4.0 | < LLOQ – 4.6 | 2.1 – 4.7 |
| Ealing | DR% (> LLOQ) | 100 | 100 | - | 100 | 100 | 62 | 100 | - | 92 |
| (13) | Mean ± SD | 3.0 ± 0.9 | 2.7 ± 0.6 | < LLOQ | 1.8 ± 0.3 | 2.9 ± 0.5 | 1.6 ± 1.4 | 2.1 ± 0.7 | < LLOQ | 3.1 ± 0.5 |
| | Median | 3.1 | 2.8 | - | 1.8 | 3.1 | 2.7 | 1.9 | - | 3.2 |
| | Range | 1.3 – 4.4 | 1.4 – 3.6 | - | 1.2 – 2.3 | 1.7 – 3.4 | < LLOQ – 4.2 | 1.1 – 3.6 | - | < LOD – 3.7 |
| Hammersmith & Fulham | DR% (> LLOQ) | 100 | 100 | - | 100 | 100 | 72 | 94 | 6 | 100 |
| (18) | Mean ± SD | 3.4 ± 0.9 | 2.9 ± 0.5 | < LLOQ | 1.7 ± 0.3 | 3.0 ± 0.5 | 2.1 ± 1.0 | 2.1 ± 0.5 | 2.7 ± 0.5 | 3.2 ± 0.7 |
| | Median | 3.5 | 3.0 | - | 1.7 | 3.1 | 2.3 | 2.1 | - | 3.1 |
| | Range | 1.9 – 4.7 | 2.0 – 3.7 | - | 1.3 – 2.2 | 1.9 – 4.0 | < LLOQ – 3.3 | < LLOQ – 3.3 | < LLOQ – 2.7 | 2.3 – 4.7 |
| Harrow | DR% (> LLOQ) | 100 | 100 | - | 100 | 100 | 100 | 100 | 50 | 100 |
| (2) | Mean ± SD | 3.2 ± 2.2 | 2.4 ± 1.5 | < LLOQ | 1.7 ± 0.8 | 2.7 ± 1.2 | 3.7 ± 2.3 | 3.0 ± 1.5 | 5.2 ± 0.3 | 3.3 ± 1.5 |
| | Median | 3.2 | 2.4 | - | 1.7 | 2.7 | 3.7 | 3.0 | - | 3.3 |
| | Range | 1.7 – 4.8 | 1.3 – 3.5 | - | 1.1 – 2.2 | 1.8 – 3.5 | 2.1 – 5.4 | 1.9 – 4.1 | < LOD – 5.2 | 2.3 – 4.3 |
| Hillingdon | DR% (> LLOQ) | 100 | 100 | - | 100 | 100 | - | 100 | - | 100 |
| (1) | Mean ± SD | 1.6 ± 0.2 | 2.0 ± 0.6 | - | 1.1 ± 0.5 | 2.3 ± 0.3 | - | 1.5 ± 0.6 | < LLOQ | 1.9 ± 0.1 |
| | Median | - | - | - | - | - | - | - | - | - |
| | Range | - | - | - | - | - | - | - | - | - |
| Richmond upon Thames | DR% (> LLOQ) | 100 | 100 | - | 100 | 100 | 100 | 100 | - | 100 |
| (3) | Mean ± SD | 3.5 ± 0.4 | 2.8 ± 0.2 | < LLOQ | 1.8 ± 0.2 | 2.9 ± 0.2 | 2.8 ± 0.4 | 2.2 ± 0.6 | < LLOQ | 4.0 ± 0.7 |
| | Median | 3.6 | 2.9 | - | 1.7 | 2.8 | 2.8 | 2.1 | - | 3.9 |
| | Range | 3.1 – 3.9 | 2.6 – 3.0 | - | 1.6 – 1.9 | 2.6 – 3.1 | 2.4 – 3.1 | 1.6 – 2.9 | - | 3.4 – 4.7 |

| | | | | | | | | | | |
|---|---|---|---|---|---|---|---|---|---|---|
| Central London | | | | | | | | | | |
| Camden | DR% (> LLOQ) | 100 | 100 | - | 100 | 100 | 100 | 100 | - | 100 |
| (1) | Mean ± SD | 4.0 ± 0.9 | 3.5 ± 0.6 | < LLOQ | 2.6 ± 0.4 | 4.0 ± 0.7 | 3.4 ± 0.2 | 3.2 ± 1.1 | < LLOQ | 3.7 ± 0.6 |
| | Median | - | - | - | - | - | - | - | - | - |
| | Range | - | - | - | - | - | - | - | - | - |
| Islington | DR% (> LLOQ) | 100 | 100 | - | 100 | 100 | 100 | 100 | - | 100 |
| (1) | Mean ± SD | 4.0 ± 0.1 | 3.3 ± 0.2 | < LLOQ | 2.3 ± 0.2 | 3.2 ± 0.1 | 2.6 ± 1.0 | 2.4 ± 0.8 | < LLOQ | 3.8 ± 0.2 |
| | Median | - | - | - | - | - | - | - | - | - |
| | Range | - | - | - | - | - | - | - | - | - |
| Kensington and Chelsea | DR% (> LLOQ) | 100 | 100 | - | 100 | 100 | 60 | 100 | - | 100 |
| (10) | Mean ± SD | 3.4 ± 0.7 | 2.7 ± 0.3 | < LLOQ | 1.7 ± 0.3 | 3.2 ± 0.4 | 1.9 ± 1.2 | 1.8 ± 0.4 | < LLOQ | 3.4 ± 0.6 |
| | Median | 3.3 | 2.7 | - | 1.8 | 3.2 | 2.3 | 1.7 | - | 3.6 |
| | Range | 2.2 – 4.3 | 2.4 – 3.3 | - | 1.3 – 2.2 | 2.5 – 3.8 | < LLOQ – 3.1 | 1.4 – 2.5 | < LOD – < LLOQ | 2.3 – 4.1 |
| Lambeth | DR% (> LLOQ)) | 100 | 100 | - | 100 | 100 | 100 | 100 | - | 100 |
| (3) | Mean ± SD | 2.8 ± 0.5 | 2.6 ± 0.2 | < LLOQ | 1.7 ± 0.4 | 2.3 ± 0.2 | 2.3 ± 0.7 | 2.1 ± 0.8 | < LLOQ | 3.6 ± 0.6 |
| | Median | 3.1 | 2.7 | - | 1.5 | 2.4 | 1.9 | 2.4 | - | 4.0 |
| | Range | 2.3 – 3.1 | 2.4 – 2.8 | - | 1.4 – 2.2 | 2.1 – 2.5 | 1.8 – 3.1 | 1.2 – 2.6 | < LOD – < LLOQ | 2.9 – 4.0 |
| Southwark | DR% (> LLOQ) | 100 | 100 | - | 100 | 100 | - | 100 | - | 100 |
| (1) | Mean ± SD | 2.3 ± 0.7 | 2.3 ± 0.2 | - | 1.6 ± 0.3 | 2.0 ± 0.5 | < LLOQ | 1.7 ± 0.1 | - | 2.5 ± 0.5 |
| | Median | - | - | - | - | - | - | - | - | - |
| | Range | - | - | - | - | - | - | - | - | - |
| Westminster | DR% (> LLOQ) | 100 | 100 | - | 100 | 100 | 71 | 86 | 17 | 100 |
| (7) | Mean ± SD | 2.8 ± 0.7 | 2.6 ± 0.6 | < LLOQ | 1.6 ± 0.3 | 2.5 ± 0.7 | 2.2 ± 1.1 | 2.0 ± 0.6 | 2.5 ± 0.5 | 3.0 ± 0.7 |
| | Median | 3.2 | 2.6 | - | 1.6 | 2.4 | 2.4 | 2.0 | - | 3.3 |
| | Range | 1.6 – 3.8 | 1.6 – 3.5 | - | 1.1 – 2.1 | 1.3 – 3.3 | < LLOQ – 3.1 | < LLOQ – 2.7 | < LOD – 2.5 | 1.8 – 3.5 |
| East London | | | | | | | | | | |
| Bexley | DR% (> LLOQ) | 100 | 100 | - | - | 50 | - | 50 | - | 50 |
| (2) | Mean ± SD | 1.6 ± 0.2 | 0.9 ± 0.3 | - | - | 1.0 ± 0.3 | - | 1.2 ± 0.3 | - | 1.1 ± 0.3 |
| | Median | 1.6 | 0.9 | - | - | - | - | - | - | - |
| | Range | 1.4 – 1.7 | 0.6 – 1.1 | - | - | < LLOQ – 1.0 | - | < LOD – 1.2 | - | < LOD – 1.1 |
| Greenwich | DR% (> LLOQ) | 100 | 100 | - | 100 | 100 | 100 | 100 | - | 100 |
| (1) | Mean ± SD | 2.6 ± 0.2 | 2.7 ± 0.3 | < LLOQ | 1.4 ± 0.1 | 1.8 ± 0.2 | 2.0 ± 1.0 | 2.1 ± 1.0 | < LLOQ | 2.9 ± 0.3 |
| | Median | - | - | - | - | - | - | - | - | - |
| | Range | - | - | - | - | - | - | - | - | - |

| | | | | | | | | | | |
|---|---|---|---|---|---|---|---|---|---|---|
| Hackney (2) | DR% (> LLOQ) | 100 | 100 | 100 | 100 | 100 | 100 | 100 | 50 | 100 |
| | Mean ± SD | 3.2 ± 0.9 | 2.7 ± 0.6 | 1.2 ± 0.01 | 2.4 ± 0.1 | 3.3 ± 0.3 | 2.7 ± 0.8 | 2.8 ± 0.1 | 2.8 ± 0.1 | 4.0 ± 1.0 |
| | Median | 3.2 | 2.7 | 1.2 | 2.4 | 3.3 | 2.7 | 2.8 | - | 4.0 |
| | Range | 2.6 – 3.8 | 2.3 – 3.2 | 1.22 –1.23 | 2.3 – 2.4 | 3.1 – 3.5 | 2.1 – 3.2 | 2.7 – 2.9 | < LOD – 2.8 | 3.3 – 4.7 |
| Lewisham (2) | DR% (> LLOQ) | 100 | 100 | - | 100 | 100 | 100 | 100 | - | 100 |
| | Mean ± SD | 2.8 ± 1.3 | 2.5 ± 1.2 | < LLOQ | 1.7 ± 0.9 | 2.7 ± 1.7 | 3.3 ± 1.8 | 2.5 ± 0.01 | < LLOQ | 3.6 ± 1.9 |
| | Median | 2.8 | 2.5 | - | 1.7 | 2.7 | 3.3 | 2.5 | - | 3.6 |
| | Range | 1.9 – 3.7 | 1.6 – 3.3 | - | 1.1 – 2.4 | 1.5 – 3.9 | 2.1 – 4.5 | 2.47 – 2.48 | < LOD – < LLOQ | 2.3 – 4.9 |
| Newham (2) | DR% (> LLOQ) | 100 | 100 | 100 | 100 | 100 | 100 | 100 | 50 | 100 |
| | Mean ± SD | 3.6 ± 0.2 | 3.3 ± 0.9 | 1.2 ± 0.3 | 2.1 ± 0.2 | 3.5 ± 0.6 | 3.0 ± 0.01 | 2.6 ± 0.2 | 2.9 ± 0.4 | 4.6 ± 0.7 |
| | Median | 3.6 | 3.3 | 1.2 | 2.2 | 3.5 | 3.0 | 2.6 | - | 4.6 |
| | Range | 3.5 – 3.7 | 2.6 – 3.9 | 1.0 – 1.4 | 2.0 – 2.3 | 3.1 – 3.9 | 3.0 – 3.01 | 2.5 – 2.7 | < LLOQ – 2.9 | 4.1 – 5.1 |
| Redbridge (1) | DR% (> LLOQ) | 100 | 100 | 100 | 100 | 100 | 100 | 100 | 100 | 100 |
| | Mean ± SD | 5.0 ± 0.2 | 4.7 ± 0.3 | 1.8 ± 0.4 | 2.5 ± 0.3 | 4.1 ± 0.4 | 3.4 ± 0.7 | 3.6 ± 0.8 | 3.2 ± 0.5 | 4.5 ± 0.6 |
| | Median | - | - | - | - | - | - | - | - | - |
| | Range | - | - | - | - | - | - | - | - | - |
| Tower Hamlets (2) | DR% (> LLOQ) | 100 | 100 | 100 | 100 | 100 | 50 | 100 | 100 | 100 |
| | Mean ± SD | 3.5 ± 0.5 | 4.0 ± 0.8 | 1.1 ± 0.02 | 2.4 ± 0.1 | 3.1 ± 0.03 | 3.9 ± 0.6 | 2.9 ± 0.9 | 3.4 ± 0.9 | 3.7 ± 0.5 |
| | Median | 3.5 | 4.0 | 1.1 | 2.3 | 3.1 | - | 2.9 | 3.4 | 3.7 |
| | Range | 3.2 – 3.9 | 3.4 – 4.5 | 1.1 – 1.14 | 2.3 – 2.4 | 3.06 – 3.1 | < LOD – 3.9 | 2.2 – 3.5 | 2.8 – 4.0 | 3.4 – 4.1 |
| Waltham Forest (2) | DR% (> LLOQ) | 100 | 100 | 100 | 100 | 100 | 100 | 100 | 100 | 100 |
| | Mean ± SD | 4.4 ± 0.4 | 4.4 ± 0.1 | 1.3 ± 0.1 | 3.0 ± 0.1 | 4.0 ± 0.5 | 3.3 ± 0.1 | 3.9 ± 0.4 | 3.2 ± 1.1 | 5.4 ± 0.8 |
| | Median | 4.4 | 4.4 | 1.3 | 3.0 | 4.0 | 3.3 | 3.9 | 3.2 | 5.4 |
| | Range | 4.1 – 4.7 | 4.3 – 4.4 | 1.2 – 1.4 | 2.9 – 3.1 | 3.6 – 4.4 | 3.2 – 3.4 | 3.6 – 4.2 | 2.4 – 4.0 | 4.9 – 6.0 |
| South London | | | | | | | | | | |
| Bromley (2) | DR% (> LLOQ) | 100 | 100 | - | 100 | 100 | 100 | 100 | - | 100 |
| | Mean ± SD | 2.5 ± 1.6 | 2.4 ± 1.4 | < LLOQ | 1.5 ± 1.0 | 2.4 ± 1.5 | 2.9 ± 1.2 | 2.2 ± 0.1 | - | 2.9 ± 1.5 |
| | Median | 2.4 | 2.4 | - | 1.5 | 2.4 | 2.9 | 2.3 | - | 2.9 |
| | Range | 1.3 – 3.6 | 1.4 – 3.4 | - | 0.8 – 2.2 | 1.3 – 3.5 | 2.1 – 3.8 | 2.2 – 2.4 | - | 1.9 – 4.0 |
| Croydon (1) | DR% (> LLOQ) | 100 | 100 | - | - | 100 | - | - | 100 | 100 |
| | Mean ± SD | 1.1 ± 0.2 | 0.9 ± 0.2 | - | < LLOQ | 1.3 ± 0.2 | < LLOQ | < LLOQ | 3.0 ± 1.2 | 3.3 ± 0.9 |
| | Median | - | - | - | - | - | - | - | - | - |
| | Range | - | - | - | - | - | - | - | - | - |

| | | | | | | | | | | |
|---|---|---|---|---|---|---|---|---|---|---|
| Kingston upon Thames | DR% (> LLOQ) | 100 | 100 | - | 100 | 100 | 100 | 100 | - | 100 |
| (1) | Mean ± SD | 2.4 ± 0.1 | 1.5 ± 0.2 | < LLOQ | 1.2 ± 0.2 | 2.1 ± 0.1 | 1.9 ± 0.2 | 1.4 ± 0.6 | - | 2.4 ± 0.8 |
| | Median | - | - | - | - | - | - | - | - | - |
| | Range | - | - | - | - | - | - | - | - | - |
| Merton | DR% (> LLOQ) | 100 | 100 | - | 50 | 100 | 50 | 100 | - | 100 |
| (2) | Mean ± SD | 2.2 ± 2.0 | 1.8 ± 1.5 | < LLOQ | 2.0 ± 0.6 | 2.1 ± 1.2 | 2.3 ± 0.6 | 2.4 ± 0.6 | < LLOQ | 2.6 ± 2.3 |
| | Median | 2.2 | 1.8 | - | - | 2.0 | - | 2.4 | - | 2.6 |
| | Range | 0.8 – 3.6 | 0.7 – 2.9 | - | < LLOQ – 2.0 | 1.2 – 2.9 | < LLOQ – 2.3 | 2.0 – 2.8 | < LOD – < LLOQ | 0.9 – 4.2 |
| Sutton | DR% (> LLOQ) | - | - | - | - | - | - | 100 | - | 100 |
| (1) | Mean ± SD | - | - | - | < LLOQ | - | < LLOQ | 1.1 ± 0.6 | < LLOQ | 1.5 ± 0.8 |
| | Median | - | - | - | - | - | - | - | - | - |
| | Range | - | - | - | - | - | - | - | - | - |
| Wandsworth | DR% (> LLOQ) | 100 | 100 | - | 100 | 100 | 67 | 100 | - | 100 |
| (3) | Mean ± SD | 2.2 ± 1.5 | 1.8 ± 0.9 | < LLOQ | 1.3 ± 0.8 | 2.1 ± 1.4 | 2.5 ± 0.6 | 2.4 ± 0.03 | - | 2.6 ± 1.9 |
| | Median | 1.4 | 1.4 | - | 0.9 | 1.3 | 2.5 | 2.4 | - | 1.6 |
| | Range | 1.4 – 3.9 | 1.2 – 2.9 | - | 0.8 – 2.3 | 1.3 – 3.8 | < LOD – 2.9 | 2.3 – 2.4 | - | 1.4 – 4.8 |
| North London | | | | | | | | | | |
| Enfield | DR% (> LLOQ) | 100 | 100 | - | 100 | 100 | 100 | 100 | - | 100 |
| (1) | Mean ± SD | 2.9 ± 0.2 | 2.0 ± 0.2 | - | 1.6 ± 0.1 | 2.3 ± 0.7 | 3.0 ± 0.8 | 2.2 ± 0.5 | - | 2.3 ± 0.5 |
| | Median | - | - | - | - | - | - | - | - | - |
| | Range | - | - | - | - | - | - | - | - | - |
| Haringey | DR% (> LLOQ) | 100 | 100 | - | 100 | 100 | 100 | 100 | - | 100 |
| (1) | Mean ± SD | 1.9 ± 0.2 | 1.3 ± 0.1 | - | 1.9 ± 0.5 | 2.3 ± 0.3 | 2.5 ± 0.3 | 2.3 ± 0.8 | - | 2.5 ± 0.9 |
| | Median | - | - | - | - | - | - | - | - | - |
| | Range | - | - | - | - | - | - | - | - | - |

DR% (> LOD) – Detection rate above the limit of detection
DR% (> LLOQ) – Detection rate above the lower limit of detection

**Supplementary Table S4.** Average removal efficiencies (n = 3) ± standard deviation of PFAS compounds for all filter jugs tested. The PFAS removal score is indicated in parenthesis.

| PFAS Compound | Jug A | Jug B | Jug C | Jug D | Jug E |
|---|---|---|---|---|---|
| 4:2 FTS | 99 ± 2 (1) | 100 ± 0 (1) | 93 ± 5 (2) | 100 ± 0.1 (1) | 98 ± 0.1 (1) |
| 5:3 FTCA | - | - | - | - | - |
| 6:2 Cl-PFESA | 99 ± 1 (1) | 100 ± 0 (1) | 94 ± 6 (2) | 100 ± 0.1 (1) | 98 ± 0.1 (1) |
| 6:2 FTS | 99 ± 2 (1) | 100 ± 0 (1) | 94 ± 5 (2) | 100 ± 0.1 (1) | 99 ± 0.2 (1) |
| 7:3 FTCA | - | - | - | - | - |
| 8:2 Cl-PFESA | 100 ± 0.7 (1) | 100 ± 0.1 (1) | 93 ± 8 (2) | 100 ± 0.1 (1) | 99 ± 0.1 (1) |
| 8:2 FTS | 100 ± 0.8 (1) | 100 ± 0 (1) | 96 ± 5 (1) | 100 ± 0.1 (1) | 99 ± 0.2 (1) |
| DONA/ADONA | 99 ± 2 (1) | 100 ± 0 (1) | 89 ± 10 (2) | 100 ± 0 (1) | 98 ± 0.1 (1) |
| HFPO-TA | - | - | - | - | - |
| L-PFDoS | 100 ± 0.3 (1) | 99 ± 0.8 (1) | 94 ± 6 (2) | 100 ± 0.5 (1) | 99 ± 0.3 (1) |
| L-PFUdS | 100 ± 0.6 (1) | 100 ± 0.3 (1) | 94 ± 7 (2) | 100 ± 0.4 (1) | 99 ± 0.2 (1) |
| MeFOSA | - | - | - | - | - |
| N-EtFOSAA | 99 ± 1.3 (1) | 100 ± 0.1 (1) | 89 ± 12 (2) | 100 ± 0 (1) | 97 ± 0.1 (1) |
| N-MeFOSAA | 99 ± 1 (1) | 100 ± 0 (1) | 93 ± 8 (2) | 100 ± 0 (1) | 98 ± 0.1 (1) |
| PFBA | 99 ± 0.7 (1) | 100 ± 0.2 (1) | 89 ± 6 (2) | 99 ± 0.7 (1) | 98 ± 0.4 (1) |
| PFBS | 99 ± 1 (1) | 100 ± 0 (1) | 92 ± 6 (2) | 100 ± 0.3 (1) | 99 ± 0 (1) |
| PFDA | 99 ± 1 (1) | 100 ± 0 (1) | 94 ± 6 (2) | 99 ± 0.8 (1) | 98 ± 0.1 (1) |
| PFDoA | 99 ± 1 (1) | 100 ± 0.1 (1) | 92 ± 9 (2) | 100 ± 0.3 (1) | 99 ± 0.4 (1) |
| PFDS | 100 ± 0.7 (1) | 100 ± 0.1 (1) | 94 ± 6 (2) | 100 ± 0.1 (1) | 99 ± 0.1 (1) |
| PFecHS | 99 ± 1 (1) | 100 ± 0 (1) | 89 ± 9 (2) | 100 ± 0 (1) | 99 ± 0.2 (1) |
| PFEESA | 99 ± 1 (1) | 100 ± 0 (1) | 90 ± 8 (2) | 100 ± 0.1 (1) | 99 ± 0 (1) |
| PFHpA | 99 ± 1 (1) | 100 ± 0 (1) | 89 ± 9 (2) | 99 ± 2 (1) | 98 ± 0.1 (1) |
| PFHpS | 98 ± 1 (1) | 99 ± 0.1 (1) | 91 ± 7 (2) | 98 ± 3 (1) | 98 ± 0.4 (1) |
| PFHxA | 99 ± 1 (1) | 100 ± 0.1 (1) | 91 ± 8 (2) | 98 ± 2 (1) | 98 ± 0.1 (1) |
| PFHxDA | 97 ± 2 (1) | 92 ± 7 (2) | 88 ± 5 (3) | 92 ± 8 (2) | 78 ± 11 (4) |
| PFHxS | 99 ± 1 (1) | 100 ± 0 (1) | 90 ± 10 (2) | 99 ± 1 (1) | 99 ± 0.1 (1) |
| PFMOBA | 100 ± 0.8 (1) | 100 ± 0 (1) | 95 ± 4 (1) | 100 ± 0.2 (1) | 98 ± 0.1 (1) |
| PFMOPrA | 100 ± 0.6 (1) | 100 ± 0 (1) | 92 ± 5 (2) | 100 ± 0 (1) | 98 ± 0.1 (1) |
| PFNA | 99 ± 2 (1) | 100 ± 0.1 (1) | 93 ± 6 (2) | 100 ± 0.6 (1) | 98 ± 0.3 (1) |
| PFNS | 99 ± 0.8 (1) | 100 ± 0 (1) | 95 ± 5 (1) | 100 ± 0.1 (1) | 99 ± 0.1 (1) |
| PFOA | 99 ± 2 (1) | 100 ± 0 (1) | 90 ± 8 (2) | 99 ± 1 (1) | 98 ± 0.3 (1) |
| PFODA | 97 ± 3 (1) | 93 ± 6 (2) | 87 ± 4 (3) | 74 ± 35 (5) | 58 ± 20.9 (8) |
| PFOS | 99 ± 1 (1) | 100 ± 0 (1) | 93 ± 6 (2) | 100 ± 0.2 (1) | 99 ± 0.1 (1) |
| PFPeA | 99 ± 0.9 (1) | 100 ± 0.1 (1) | 95 ± 3 (1) | 100 ± 0.4 (1) | 98 ± 0.1 (1) |
| PFPeS | 9 9 ± 1 (1) | 100 ± 0 (1) | 91 ± 7 (2) | 100 ± 0.2 (1) | 99 ± 0.1 (1) |
| PFTeDA | 99 ± 0.5 (1) | 98 ± 2 (1) | 94 ± 5 (2) | 99 ± 0.8 (1) | 96 ± 0.7 (1) |
| PFTrDA | 99 ± 1 (1) | 99 ± 0.5 (1) | 92 ± 9 (2) | 99 ± 0.7 (1) | 98 ± 0.2 (1) |
| PFUdA | 99 ± 1 (1) | 100 ± 0 (1) | 94 ± 7 (2) | 99 ± 0.7 (1) | 98 ± 0.2 (1) |

- unable to assess recovery

**Supplementary Table S5.** Average PFAS concentration in London tap water and average concentration in tap water after filtration.

| | | PFBA | PFBS | PFecHS | PFHpA | PFHxA | PFHxS | PFOA | PFOS | PFPeA |
|---|---|---|---|---|---|---|---|---|---|---|
| [PFAS London] | | 3.1 ± 1.0 | 2.7 ± 0.8 | 1.3 ± 0.3 | 1.8 ± 0.5 | 2.8 ± 0.8 | 2.7 ± 0.7 | 2.2 ± 0.7 | 3.3 ± 0.9 | 3.3 ± 1.0 |
| | 20 L | - | - | - | - | - | - | < LLOQ | - | < LLOQ |
| | 40 L | - | - | - | - | - | - | < LLOQ | - | - |
| Site 1 | 60 L | - | < LLOQ | - | < LLOQ | - | - | 0.3 ± 0.1 | - | < LLOQ |
| | 80 L | - | - | - | - | - | - | < LLOQ | - | - |
| | 100 L | < LLOQ | - | - | - | - | - | < LLOQ | - | - |
| | 20 L | - | - | - | - | - | - | < LLOQ | - | - |
| | 40 L | - | - | - | < LLOQ | < LLOQ | - | 0.2 ± 0.02 | - | - |
| Site 2 | 60 L | - | - | - | - | - | - | 0.2 ± 0.1 | - | - |
| | 80 L | - | - | - | - | - | - | < LLOQ | - | - |
| | 100 L | - | - | - | - | - | - | < LLOQ | - | - |

- not detected,

< LLOQ: below limit if quantification, refer to **Supplementary Table S9** for individual values

**Supplementary Table S6.** Comparison between calibration lines and the quantification results of QCs (average ± standard deviation) prepared using the Andrew+ robot and the laboratory user for all PFAS compounds. The mean absolute error (MAE) between the slope, intercept and QC quantification values is presented.

| Compound | Andrew+ | | | | | | Laboratory user | | | | | | MAE | | | |
|---|---|---|---|---|---|---|---|---|---|---|---|---|---|---|---|---|
| | Range ($n \geq 5$) | Linearity ($R^2$) | Slope | Intercept | 10 ng/L[a,c] ($n = 7$) | 10 ng/L[b,c] ($n = 7$) | Range ($n \geq 5$) | Linearity ($R^2$) | Slope | Intercept | 10 ng/L[a,d] ($n = 7$) | 10 ng/L[b,d] ($n = 7$) | Slope | Intercept | 10 ng/L[a] | 10 ng/L[b] |
| 4-2FTS* | 0.5 - 100 | 1.00 | 0.99 | -1.29 | 11 ± 0.3 | 10 ± 0.4 | 0.5 - 100 | 1.00 | 1.05 | -1.39 | 12 ± 0.5 | 13 ± 0.4 | 0.06 | 0.1 | 0.7 | 0.9 |
| 5:3 FTCA | 0.5 - 100 | 1.00 | 1.04 | 2.91 | 11 ± 0.5 | 10 ± 0.4 | 0.5 - 100 | 1.00 | 1.07 | 2.83 | 12 ± 0.5 | 13 ± 0.6 | 0.03 | 0.08 | 0.9 | 1.0 |
| 6:2 Cl-PFESA | 0.5 - 100 | 1.00 | 1.04 | 3.65 | 11 ± 0.4 | 9 ± 0.7 | 0.5 - 100 | 1.00 | 1.01 | 3.67 | 9 ± 0.7 | 11 ± 0.4 | 0.03 | 0.02 | 1.4 | 1.4 |
| 6-2FTS* | 0.5 - 100 | 0.99 | 1.04 | -0.47 | 11 ± 1.3 | 9 ± 0.7 | 0.5 - 100 | 0.99 | 0.98 | -0.42 | 9 ± 0.7 | 11 ± 1 | 0.06 | 0.05 | 1.7 | 1.6 |
| 7:3 FTCA | 0.5 - 100 | 0.99 | 0.99 | 2.93 | 8 ± 0.6 | 8 ± 0.6 | 0.5 - 100 | 0.97 | 1.22 | 2.52 | 17 ± 1 | 16 ± 1 | 0.23 | 0.41 | 0.2 | 0.4 |
| 8:2Cl-PFESA | 0.5 - 100 | 0.99 | 1.31 | 2.43 | 23 ± 4 | 14 ± 2 | 1 - 75 | 0.97 | 1.20 | 2.55 | 12 ± 2 | 19 ± 3 | 0.11 | 0.12 | 8.9 | 7.5 |
| 8-2FTS* | 0.5 - 100 | 0.98 | 1.05 | -0.68 | 11 ± 3 | 9 ± 2 | 0.5 - 75 | 0.98 | 1.06 | -0.78 | 11 ± 2 | 13 ± 4 | 0.01 | 0.1 | 1.8 | 2.2 |
| DONA | 0.5 - 100 | 1.00 | 1.00 | 4.03 | 10 ± 0.4 | 9 ± 0.4 | 0.5 - 100 | 1.00 | 1.00 | 4.00 | 10 ± 0.5 | 10 ± 0.4 | 0 | 0.03 | 0.4 | 0.5 |
| HFPO-TA | 5 - 100 | 0.99 | 1.22 | 1.33 | 19 ± 4 | 18 ± 3 | 2.5 - 100 | 0.96 | 1.04 | 1.61 | 11 ± 2 | 11 ± 3 | 0.18 | 0.28 | 1.0 | 0.6 |
| L-PFDoS | 5 - 75 | 0.92 | 1.00 | 1.78 | 6 ± 3 | 5 ± 3 | 2.5 - 75 | 0.93 | 1.05 | 1.52 | 9 ± 6 | 11 ± 6 | 0.05 | 0.26 | 0.9 | 1.6 |
| L-PFUdS | 2.5 - 100 | 0.98 | 1.31 | 1.42 | 16 ± 6 | 11 ± 3 | 0.5 - 75 | 0.98 | 1.39 | 1.12 | 22 ± 5 | 31 ± 11 | 0.08 | 0.3 | 4.8 | 9.1 |
| MeFOSA | 2.5 - 75 | 0.62 | 0.82 | 1.75 | 10 ± 3 | 10 ± 2 | 2.5 - 75 | 0.66 | 0.71 | 1.94 | 8 ± 1 | 7 ± 3 | 0.11 | 0.19 | 0.6 | 0.5 |
| N-EtFOSAA* | 2.5 - 100 | 0.97 | 1.24 | -1.72 | 22 ± 3 | 17 ± 2 | 1 - 100 | 0.95 | 1.15 | -1.62 | 15 ± 2 | 18 ± 3 | 0.09 | 0.1 | 4.5 | 3.7 |
| N-MeFOSAA* | 1 - 100 | 0.98 | 1.07 | -0.54 | 10 ± 3 | 8 ± 2 | 1 - 100 | 0.98 | 1.23 | -0.87 | 14 ± 4 | 19 ± 5 | 0.16 | 0.33 | 2.4 | 4.4 |
| PFBA | 0.5 – 100 | 0.99 | 1.29 | 2.86 | 19 ± 3 | 19 ± 3 | 0.5 – 100 | 1.00 | 1.09 | 3.14 | 12 ± 2 | 12 ± 2 | 0.20 | 0.28 | 0.3 | 0.2 |
| PFBS* | 0.5 - 100 | 0.98 | 1.18 | -0.83 | 15 ± 1 | 14 ± 2 | 0.5 - 100 | 1.00 | 1.05 | -0.67 | 11 ± 2 | 12 ± 0.9 | 0.13 | 0.16 | 0.8 | 0.6 |
| PFDA* | 1 - 100 | 0.99 | 0.96 | -0.47 | 10 ± 1 | 9 ± 0.6 | 0.5 - 75 | 0.96 | 1.17 | -0.76 | 14 ± 1 | 15 ± 2 | 0.21 | 0.29 | 1.0 | 1.6 |
| PFDoA* | 5 - 100 | 0.95 | 1.14 | -0.94 | 23 ± 4 | 20 ± 5 | 1 - 75 | 0.96 | 0.99 | -0.71 | 14 ± 4 | 16 ± 3 | 0.15 | 0.23 | 3.1 | 2.1 |
| PFDS | 1 - 100 | 0.93 | 1.19 | 2.00 | 13 ± 3 | 10 ± 2 | 0.5 - 100 | 0.98 | 1.17 | 1.95 | 12 ± 3 | 15 ± 4 | 0.02 | 0.05 | 2.8 | 3.2 |
| PFecHS | 0.5 - 100 | 1.00 | 1.02 | 3.66 | 10 ± 0.3 | 10 ± 0.5 | 0.5 - 100 | 1.00 | 1.02 | 3.62 | 10 ± 0.5 | 11 ± 0.4 | 0 | 0.04 | 0.4 | 0.4 |
| PFEESA | 0.5 - 100 | 1.00 | 1.01 | 3.85 | 10 ± 0.4 | 10 ± 0.6 | 0.5 - 100 | 1.00 | 1.00 | 3.83 | 10 ± 0.6 | 10 ± 0.5 | 0.01 | 0.02 | 0.2 | 0.2 |
| PFHpA* | 1 - 100 | 1.00 | 1.04 | -1.38 | 11 ± 0.3 | 10 ± 0.8 | 0.5 - 100 | 0.99 | 1.08 | -1.47 | 12 ± 1 | 13 ± 0.4 | 0.04 | 0.09 | 1.0 | 1.2 |
| PFHpS | 0.5 - 100 | 0.99 | 1.16 | 2.64 | 14 ± 2 | 13 ± 0.7 | 1 - 100 | 0.99 | 1.15 | 2.62 | 14 ± 0.7 | 15 ± 2 | 0.01 | 0.02 | 0.7 | 0.8 |
| PFHxA | 0.5 - 100 | 1.00 | 1.00 | 3.40 | 10 ± 0.5 | 10 ± 0.8 | 0.5 - 100 | 0.99 | 0.99 | 3.38 | 10 ± 0.8 | 11 ± 0.6 | 0.01 | 0.02 | 0.4 | 0.5 |
| PFHxDA* | 10 - 100 | 0.99 | 1.07 | -1.47 | 15 ± 3 | 14 ± 2 | 0.5 - 75 | 0.99 | 0.99 | -1.45 | 14 ± 2 | 16 ± 3 | 0.08 | 0.02 | 1.8 | 1.8 |
| PFHxS* | 5 – 100 | 0.99 | 0.96 | -0.72 | 9 ± 2 | 7 ± 0.9 | 1 -100 | 0.99 | 1.05 | -0.86 | 9 ± 1 | 11 ± 2 | 0.09 | 0.14 | 0.5 | 3.5 |
| PFMOBA | 0.5 - 100 | 1.00 | 1.01 | 3.29 | 10 ± 0.2 | 10 ± 0.6 | 0.5 - 100 | 1.00 | 1.03 | 3.24 | 11 ± 0.7 | 11 ± 0.2 | 0.02 | 0.05 | 0.4 | 0.4 |
| PFMOPrA | 0.5 - 100 | 1.00 | 1.05 | 2.90 | 11 ± 0.6 | 10 ± 0.6 | 0.5 - 100 | 1.00 | 1.04 | 2.88 | 11 ± 0.6 | 11 ± 0.6 | 0.01 | 0.02 | 0.4 | 0.4 |
| PFNA* | 2.5 - 100 | 1.00 | 1.00 | -1.35 | 11 ± 0.6 | 10 ± 0.9 | 1 - 100 | 0.99 | 1.01 | -1.40 | 11 ± 1 | 12 ± 0.6 | 0.01 | 0.05 | 1.0 | 1.2 |
| PFNS | 0.5 - 100 | 0.99 | 1.27 | 2.27 | 17 ± 3 | 15 ± 2 | 0.5 - 100 | 0.97 | 1.08 | 2.52 | 10 ± 1 | 12 ± 2 | 0.19 | 0.25 | 2.1 | 1.4 |
| PFOA* | 5 - 100 | 0.99 | 1.00 | -0.62 | 12 ± 0.9 | 10 ± 0.9 | 0.5 - 100 | 0.99 | 0.87 | -0.47 | 9 ± 0.8 | 10 ± 0.7 | 0.13 | 0.15 | 1.8 | 1.5 |
| PFODA | 1 - 100 | 0.97 | 1.20 | 2.65 | 12 ± 1 | 16 ± 2 | 1 - 75 | 0.97 | 0.85 | 3.10 | 8 ± 0.7 | 6 ± 0.5 | 0.35 | 0.45 | 4.1 | 2.0 |
| PFOS* | 2.5 – 100 | 0.98 | 1.03 | -0.62 | 8 ± 1 | 7 ± 1 | 1 – 100 | 0.97 | 1.15 | -0.81 | 9 ± 2 | 12 ± 2 | 0.12 | 0.19 | 0.9 | 4.8 |
| PFPeA | 0.5 - 100 | 1.00 | 1.10 | 3.13 | 14 ± 0.9 | 13 ± 0.9 | 0.5 - 100 | 0.99 | 0.99 | 3.27 | 10 ± 0.7 | 11 ± 0.7 | 0.11 | 0.14 | 1.0 | 0.8 |
| PFPeS | 0.5 - 100 | 0.99 | 1.07 | 2.94 | 11 ± 0.8 | 11 ± 1 | 0.5 - 100 | 1.00 | 1.03 | 2.97 | 11 ± 0.9 | 11 ± 0.8 | 0.04 | 0.03 | 0.3 | 0.3 |

| | | | | | | | | | | | | | | | | |
|---|---|---|---|---|---|---|---|---|---|---|---|---|---|---|---|---|
| PFTeDA* | 5 - 100 | 0.66 | 0.73 | -0.90 | 13 ± 6 | 14 ± 7 | 10 - 75 | 0.30 | 0.38 | -0.48 | 10 ± 5 | 10 ± 5 | 0.35 | 0.42 | 1.1 | 0.8 |
| PFTrDA | 1 - 75 | 0.81 | 0.71 | 3.10 | 7 ± 1 | 8 ± 2 | 0.5 - 75 | 0.91 | 0.82 | 2.94 | 10 ± 3 | 8 ± 2 | 0.11 | 0.16 | 1.5 | 1.9 |
| PFUdA* | 0.5 - 100 | 0.96 | 0.94 | -1.41 | 10 ± 0.7 | 10 ± 1 | 1 - 100 | 0.96 | 1.03 | -1.63 | 15 ± 2 | 15 ± 1 | 0.09 | 0.22 | 0.3 | 0.5 |

* Corresponding SIL-IS analogue used for peak area ratio-based assessment.
[a]: 10 ng/L QC prepared by the Andrew + robot.
[b]: 10 ng/L QC prepared by the Laboratory user.
[c]: Quantified using the calibration prepared by the Andrew+ robot.
[d]: Quantified using the calibration prepared by the Laboratory user.

**Supplementary Table S7.** Method performance characteristics for samples prepared in laboratory tap water, analysed via direct injection. LOD: limit of detection; LLOQ: Lower limit of quantification.

| Compound | Range ($n \geq 5$) | Linearity ($R^2$) | LOD (ng/L) | LLOQ (ng/L) | Peak Area precision ($n$ = 7) | | Matrix Effects | | Inaccuracy ($n$ = 7) | |
|---|---|---|---|---|---|---|---|---|---|---|
| | | | | | 5 ng/L | 10 ng/L | 5 ng/L | 10 ng/L | 5 ng/L | 10 ng/L |
| 4:2 FTS* | 2.0 - 75 | 0.99 | 0.6 | 2.0 | 15 | 12 | -2 | 23 | 16 | 11 |
| 5:3 FTCA | 0.9 - 100 | 0.99 | 0.3 | 0.9 | 14 | 16 | 3 | 15 | -6 | -4 |
| 6:2 Cl-PFESA | 1 - 50 | 0.97 | 0.3 | 1.0 | 25 | 25 | -34 | 19 | -4 | 25 |
| 6:2 FTS* | 1.5 - 100 | 1.00 | 0.4 | 1.5 | 15 | 10 | 3 | 36 | -5 | -12 |
| 7:3 FTCA | 1.2 - 100 | 0.99 | 0.4 | 1.2 | 29 | 14 | -7 | 21 | -10 | 4 |
| 8:2 Cl-PFESA | 2.1 - 50 | 0.91 | 0.6 | 2.1 | 50 | 29 | -58 | 20 | -37 | -31 |
| 8:2 FTS* | 3.4 - 75 | 0.99 | 1.0 | 3.4 | 51 | 26 | -37 | 22 | 29 | 43 |
| DONA | 0.4 - 100 | 1.00 | 0.1 | 0.4 | 10 | 11 | 4 | 16 | 1 | 14 |
| HFPO-TA | 1.2 - 75 | 0.97 | 0.4 | 1.2 | 35 | 23 | -1 | 24 | -7 | 14 |
| L-PFDoS | 3.6 - 50 | 0.24 | 1.1 | 3.6 | 40 | 71 | 80 | 97 | -28 | -61 |
| L-PFUdS | 2.0 - 50 | 0.78 | 0.6 | 2.0 | 41 | 46 | -55 | 33 | -28 | -42 |
| MeFOSA | 2.1 - 75 | 0.99 | 0.6 | 2.1 | 65 | 22 | 178 | 108 | 22 | 4 |
| N-EtFOSAA* | 4.0 - 75 | 0.99 | 1.2 | 4.0 | 58 | 38 | -52 | 19 | 82 | 46 |
| N-MeFOSAA* | 6.6 - 75 | 0.99 | 2.0 | 6.6 | 28 | 33 | -46 | 24 | 103 | 109 |
| PFBA | 0.6 – 100 | 0.99 | 0.2 | 0.6 | 16 | 11 | 119 | 21 | 26 | 3 |
| PFBS* | 0.6 - 100 | 1.00 | 0.2 | 0.6 | 13 | 8 | 53 | 14 | 30 | 11 |
| PFDA* | 2.2 - 100 | 1.00 | 0.7 | 2.2 | 55 | 18 | -30 | 20 | 2 | -7 |
| PFDoA* | 3.4 - 75 | 0.88 | 1.0 | 3.4 | 61 | 38 | -9 | 42 | 17 | -21 |
| PFDS | 6.2 - 75 | 0.80 | 1.9 | 6.2 | 29 | 54 | -46 | 34 | 33 | 68 |
| PFecHS | 0.7 - 100 | 0.99 | 0.2 | 0.7 | 10 | 14 | 1 | 13 | 21 | 33 |
| PFEESA | 0.3 - 100 | 1.00 | 0.1 | 0.3 | 13 | 7 | 1 | 11 | -1 | 13 |
| PFHpA* | 0.8 - 100 | 1.00 | 0.2 | 0.8 | 13 | 9 | 29 | 29 | 23 | 1 |
| PFHpS | 1.0 - 75 | 0.97 | 0.3 | 1.0 | 38 | 18 | -12 | 24 | -27 | 2 |
| PFHxA | 0.8 - 100 | 0.99 | 0.3 | 0.8 | 20 | 14 | 60 | 21 | 39 | 17 |
| PFHxDA* | 2.0 - 75 | 1.00 | 0.6 | 2.0 | 0 | 0 | 21 | 57 | 36 | -4 |
| PFHxS* | 1.8 - 100 | 1.00 | 0.6 | 1.8 | 18 | 9 | 54 | 26 | 137 | 81 |
| PFMOBA | 0.5 - 100 | 0.99 | 0.2 | 0.5 | 16 | 13 | 5 | 14 | 12 | 8 |
| PFMOPrA | 0.5 - 100 | 0.99 | 0.2 | 0.5 | 13 | 12 | 17 | 14 | -3 | -6 |
| PFNA* | 1.1 - 100 | 0.99 | 0.3 | 1.1 | 19 | 12 | 0 | 22 | 1 | -17 |
| PFNS | 4.0 - 75 | 0.97 | 1.2 | 4.0 | 55 | 36 | -33 | 36 | -18 | 0 |
| PFOA* | 1.0 - 100 | 1.00 | 0.3 | 1.0 | 19 | 9 | 32 | 21 | 25 | -17 |
| PFODA | 1.1 - 50 | 0.96 | 0.3 | 1.1 | 30 | 20 | 43 | 40 | -24 | -36 |
| PFOS* | 2.4 - 100 | 0.99 | 0.7 | 2.4 | 20 | 34 | 21 | 33 | 45 | 13 |
| PFPeA | 0.6 - 75 | 0.96 | 0.2 | 0.6 | 10 | 8 | 49 | 18 | 35 | 2 |
| PFPeS | 1.0 - 100 | 0.99 | 0.3 | 1.0 | 24 | 17 | 11 | 32 | -17 | -3 |
| PFTeDA* | 41 - 100 | 0.02 | 12.3 | 41.0 | 55 | 48 | -16 | 49 | 844 | 585 |
| PFTrDA | 2.5 - 75 | 0.46 | 0.7 | 2.5 | 49 | 48 | -3 | 31 | 14 | -49 |
| PFUdA* | 2.1 - 75 | 0.98 | 0.6 | 2.1 | 36 | 36 | -42 | 36 | -0.1 | -28 |

* Corresponding SIL-IS analogue used for peak area ratio-based assessment.

**Supplementary Table S8. Method performance characteristics for samples prepared in laboratory HPLC-grade water, analysed via direct injection.**

| Compound | Range ($n \geq 5$) | Linearity ($R^2$) | LOD (ng/L) | LLOQ (ng/L) | Peak Area precision % ($n$ = 7) | | Inaccuracy % ($n$ = 7) | |
|---|---|---|---|---|---|---|---|---|
| | | | | | 5 ng/L | 10 ng/L | 5 ng/L | 10 ng/L |
| 4:2 FTS* | 2.4 - 100 | 0.99 | 0.7 | 2.4 | 30 | 26 | 17 | 25 |
| 5:3 FTCA | 0.7 - 100 | 0.99 | 0.2 | 0.7 | 28 | 10 | -26 | -23 |
| 6:2 Cl-PFESA | 0.7 - 75 | 0.99 | 0.2 | 0.7 | 22 | 11 | -25 | -26 |
| 6:2 FTS* | 0.8 - 100 | 0.99 | 0.2 | 0.8 | 23 | 15 | -4 | -1 |
| 7:3 FTCA | 1.2 - 100 | 0.99 | 0.4 | 1.2 | 30 | 20 | -36 | -36 |
| 8:2 Cl-PFESA | 1.0 - 75 | 0.91 | 0.3 | 1.0 | 50 | 26 | -42 | -49 |
| 8:2 FTS* | 2.9 - 100 | 0.99 | 0.9 | 2.9 | 36 | 26 | -2 | 5 |
| DONA | 0.3 - 100 | 0.99 | 0.1 | 0.3 | 20 | 9 | -16 | -23 |
| HFPO-TA | 10.4 - 75 | 0.99 | 3.1 | 10.4 | 49 | 30 | 412 | 426 |
| L-PFDoS | 4.7 – 75 | 0.74 | 1.4 | 4.7 | 46 | 61 | -17 | -27 |
| L-PFUdS | 2.3 - 75 | 0.91 | 0.7 | 2.3 | 48 | 60 | -37 | -98 |
| MeFOSA | 3.5 - 75 | 0.97 | 1.1 | 3.5 | 49 | 57 | -1 | -34 |
| N-EtFOSAA* | 1.7 - 100 | 0.99 | 0.5 | 1.7 | 33 | 23 | -10 | -20 |
| N-MeFOSAA* | 2.0 - 100 | 1.00 | 0.6 | 2.0 | 29 | 24 | -6 | 0 |
| PFBA | 1.2 - 100 | 0.98 | 0.4 | 1.2 | 26 | 12 | -17 | -17 |
| PFBS* | 0.9 - 100 | 0.99 | 0.3 | 0.9 | 15 | 9 | 17 | 10 |
| PFDA* | 1.2 - 100 | 0.97 | 0.4 | 1.2 | 21 | 21 | -12 | -29 |
| PFDoA* | 1.8 - 100 | 0.93 | 0.5 | 1.8 | 47 | 32 | -46 | -40 |
| PFDS | 0.8 - 75 | 0.94 | 0.2 | 0.8 | 71 | 44 | -79 | -72 |
| PFecHS | 0.7 - 100 | 0.99 | 0.2 | 0.7 | 13 | 11 | 3 | 0 |
| PFEESA | 0.3 - 100 | 1.00 | 0.1 | 0.3 | 16 | 9 | -17 | -21 |
| PFHpA* | 0.7 - 100 | 0.99 | 0.2 | 0.7 | 17 | 17 | -11 | -30 |
| PFHpS | 1.1 - 100 | 0.99 | 0.3 | 1.1 | 37 | 20 | -40 | -41 |
| PFHxA | 0.9 – 100 | 0.98 | 0.3 | 0.9 | 25 | 15 | -19 | -28 |
| PFHxDA* | 2.5 - 75 | 0.94 | 0.7 | 2.5 | 49 | 54 | -3 | -22 |
| PFHxS* | 1.6 – 100 | 0.99 | 0.5 | 1.6 | 44 | 22 | -13 | -13 |
| PFMOBA | 0.6 - 100 | 0.99 | 0.2 | 0.6 | 18 | 14 | -4 | -7 |
| PFMOPrA | 0.8 - 100 | 1.00 | 0.2 | 0.8 | 29 | 29 | -48 | -50 |
| PFNA* | 1.4 - 100 | 0.99 | 0.4 | 1.4 | 28 | 20 | -3 | -24 |
| PFNS | 0.8 - 75 | 0.99 | 0.2 | 0.8 | 40 | 30 | -58 | -54 |
| PFOA* | 0.8 - 100 | 1.00 | 0.2 | 0.8 | 21 | 18 | -24 | -34 |
| PFODA | 1.7 – 75 | 0.93 | 0.5 | 1.7 | 41 | 19 | 8 | -26 |
| PFOS* | 2.8 – 100 | 0.96 | 0.9 | 2.8 | 35 | 25 | 42 | 53 |
| PFPeA | 0.5 - 100 | 0.98 | 0.1 | 0.5 | 29 | 18 | -35 | -45 |
| PFPeS | 1.1 - 100 | 0.98 | 0.3 | 1.1 | 30 | 17 | -22 | -29 |
| PFTeDA* | 17.5 - 100 | 0.02 | 5.3 | 17.5 | 65 | 54 | 578 | 380 |
| PFTrDA | 0.9 - 75 | 0.86 | 0.3 | 0.9 | 43 | 31 | -35 | -51 |
| PFUdA* | 1.1 - 75 | 0.94 | 0.3 | 1.1 | 35 | 24 | -27.6 | -45 |

* Corresponding SIL-IS analogue used for peak area ratio-based assessment.

**Supplementary Table S9.** Method performance characteristics for samples prepared in HPLC-grade water using the evaporation concentration method.

| Compound | Range ($n \geq 5$) | Linearity ($R^2$) | LOD (ng/L) | LLOQ (ng/L) | Peak Area precision ($n$ = 3) | | Inaccuracy ($n$ = 3) | |
|---|---|---|---|---|---|---|---|---|
| | | | | | 5 ng/L | 10 ng/L | 5 ng/L | 10 ng/L |
| 4-2FTS | 0.4 - 100 | 0.99 | 0.1 | 0.4 | 12 | 11 | 18 | 6 |
| 5:3 FTCA | 0.7 - 100 | 0.99 | 0.2 | 0.7 | 26 | 27 | 23 | 55 |
| 6:2 Cl-PFESA | 0.7 - 100 | 1.00 | 0.2 | 0.7 | 45 | 6 | 47 | 2 |
| 6-2FTS | 0.5 - 100 | 1.00 | 0.2 | 0.5 | 24 | 17 | 13 | 39 |
| 7:3 FTCA | 3.6 - 100 | 0.90 | 1.1 | 3.6 | 75 | 52 | 88 | -11 |
| 8:2Cl-PFESA | 0.5 - 100 | 1.00 | 0.1 | 0.5 | 56 | 8 | 63 | 7 |
| 8-2FTS | 0.3 - 100 | 1.00 | 0.1 | 0.3 | 18 | 9 | 24 | 3 |
| DONA | 0.7 - 100 | 0.99 | 0.2 | 0.7 | 34 | 17 | 22 | 46 |
| HFPO-TA | 1.2 - 100 | 0.76 | 0.4 | 1.2 | 27 | 41 | -8 | -10 |
| L-PFDoS | 0.5 - 100 | 1.00 | 0.2 | 0.5 | 40 | 7 | 45 | 15 |
| L-PFUdS | 0.7 - 100 | 1.00 | 0.2 | 0.7 | 39 | 13 | 38 | 9 |
| MeFOSA | 9.4 - 75 | 0.33 | 2.8 | 9.4 | 40 | 17 | 263 | 28 |
| N-EtFOSAA | 0.7 - 100 | 1.00 | 0.2 | 0.7 | 24 | 10 | 28 | 8 |
| N-MeFOSAA | 0.6 - 100 | 0.99 | 0.2 | 0.6 | 26 | 8 | 44 | 19 |
| PFBA | 1.2 - 50 | 0.99 | 0.4 | 1.2 | 15 | 7 | 69 | 62 |
| PFBS | 0.6 - 100 | 1.00 | 0.2 | 0.6 | 34 | 6 | 29 | 8 |
| PFDA | 0.8 - 100 | 1.00 | 0.2 | 0.8 | 17 | 6 | 26 | -2 |
| PFDoA | 0.3 - 100 | 1.00 | 0.1 | 0.3 | 22 | 11 | 19 | -8 |
| PFDS | 0.7 - 100 | 1.00 | 0.2 | 0.7 | 45 | 13 | 77 | 28 |
| PFecHS | 1.1 - 100 | 0.99 | 0.3 | 1.1 | 37 | 16 | 17 | 32 |
| PFEESA | 0.9 - 100 | 1.00 | 0.3 | 0.9 | 31 | 9 | 19 | 10 |
| PFHpA | 0.6 - 100 | 0.99 | 0.2 | 0.6 | 26 | 20 | -7 | 8 |
| PFHpS | 1.0 - 100 | 0.99 | 0.3 | 1.0 | 45 | 17 | 47 | 46 |
| PFHxA | 0.9 - 100 | 1.00 | 0.3 | 0.9 | 20 | 9 | 5 | -14 |
| PFHxDA | 0.4 - 100 | 0.99 | 0.1 | 0.4 | 35 | 16 | 11 | -25 |
| PFHxS | 1.6 - 100 | 0.99 | 0.5 | 1.6 | 35 | 23 | 29 | 25 |
| PFMOBA | 0.9 - 100 | 1.00 | 0.3 | 0.9 | 17 | 7 | 14 | 10 |
| PFMOPrA | 0.4 - 100 | 1.00 | 0.1 | 0.4 | 20 | 0 | 0 | 1 |
| PFNA | 0.4 - 100 | 1.00 | 0.1 | 0.4 | 31 | 8 | 19 | 13 |
| PFNS | 0.5 - 100 | 0.99 | 0.2 | 0.5 | 40 | 11 | 63 | 23 |
| PFOA | 0.2 - 100 | 1.00 | 0.05 | 0.2 | 30 | 17 | 16 | 43 |
| PFODA | 0.8 - 100 | 0.99 | 0.3 | 0.8 | 25 | 30 | 68 | -1 |
| PFOS | 0.7 - 100 | 0.99 | 0.2 | 0.7 | 36 | 21 | 47 | 38 |
| PFPeA | 0.4 - 100 | 1.00 | 0.1 | 0.4 | 27 | 6 | 13 | 1 |
| PFPeS | 1.0 - 100 | 0.99 | 0.3 | 1.0 | 40 | 12 | 40 | 13 |
| PFTeDA | 0.6 - 100 | 1.00 | 0.2 | 0.6 | 35 | 10 | 35 | 0 |
| PFTrDA | 0.5 - 100 | 0.99 | 0.2 | 0.5 | 40 | 20 | 3 | -20 |
| PFUdA | 0.8 - 100 | 1.00 | 0.2 | 0.8 | 27 | 12 | 23 | -6 |

**Supplementary References**


1 L. Rosenblum and S. C. Wendelken, *METHOD 533: DETERMINATION OF PER- AND POLYFLUOROALKYL SUBSTANCES IN DRINKING WATER BY ISOTOPE DILUTION ANION EXCHANGE SOLID PHASE EXTRACTION AND LIQUID CHROMATOGRAPHY/TANDEM MASS SPECTROMETRY*, 2019.

2 B. Magnusson and U. Örnemark, Eds., *Eurachem Guide: The Fitness for Purpose of Analytical Methods – A Laboratory Guide to Method Validation and Related Topics*, 2nd edn., 2014.

3 J. Thompson, G. Eaglesham and J. Mueller, *Chemosphere*, 2011, **83**, 1320–1325.

4 L. Hua and W. A. Donald, *Chemosphere*, 2025, **385**, 144611.

5 J. López-Vázquez, R. Montes, R. Rodil, R. Cela, J. Á. Martínez-Pontevedra, M. T. Pena and J. B. Quintana, *Environmental Science and Pollution Research*, 2024, 1–12.

6 F. Cappelli, Y. Ait Bamai, K. Van Hoey, D. H. Kim and A. Covaci, *Environ. Res.*, 2024, **260**, 119753.

7 S. Ullah, T. Alsberg and U. Berger, *J. Chromatogr. A*, 2011, **1218**, 6388–6395.

8 T. G. Schwanz, M. Llorca, M. Farré and D. Barceló, *Science of The Total Environment*, 2016, **539**, 143–152.

9 H. A. Kaboré, S. Vo Duy, G. Munoz, L. Méité, M. Desrosiers, J. Liu, T. K. Sory and S. Sauvé, *Science of The Total Environment*, 2018, **616–617**, 1089–1100.

10 L. M. Yim, S. Taniyasu, L. W. Y. Yeung, G. Lu, L. Jin, Y. Yang, P. K. S. Lam, K. Kannan and N. Yamashita, *Environ. Sci. Technol.*, 2009, **43**, 4824–4829.

11 G. Munoz, M. Liu, S. Vo Duy, J. Liu and S. Sauvé, *Water Res.*, 2023, **233**, 119750.

12 T. Teymoorian, G. Munoz and S. Sauvé, *Environ. Int.*, 2025, **195**, 109250.

13 K. Y. Tan, G. H. Lu, H. T. Piao, S. Chen, X. C. Jiao, N. Gai, E. Yamazaki, N. Yamashita, J. Pan and Y. L. Yang, *Bull. Environ. Contam. Toxicol.*, 2017, **99**, 224–231.

14 R. Chen, G. Li, Y. Yu, X. Ma, Y. Zhuang, H. Tao and B. Shi, *Science of The Total Environment*, 2019, **697**, 134162.

15 J. Ao, T. Yuan, H. Xia, Y. Ma, Z. Shen, R. Shi, Y. Tian, J. Zhang, W. Ding, L. Gao, X. Zhao and X. Yu, *Environmental Pollution*, 2019, **254**, 112873.

16 L. N. Xie, X. C. Wang, X. J. Dong, L. Q. Su, H. J. Zhu, C. Wang, D. P. Zhang, F. Y. Liu, S. S. Hou, B. Dong, G. Q. Shan, X. Zhang and Y. Zhu, *Environ. Int.*, 2021, **146**, 106166.

17 A. He, Y. Lu, F. Chen, F. Li, K. Lv, H. Cao, Y. Sun, Y. Liang, J. Li, L. Zhao, X. Zhang, L. Li, Y. Wang and G. Jiang, *Science of The Total Environment*, 2022, **831**, 154988.

18 C. Gao, D. S. Drage, M. A. E. Abdallah, F. Quan, K. Zhang, S. Hu, X. Zhao, Y. Zheng, S. Harrad and W. Qiu, *ACS ES and T Water*, DOI:10.1021/ACSESTWATER.4C00533/ASSET/IMAGES/LARGE/EW4C00533_0004.JPEG.

19 Y. Q. Wang, L. X. Hu, T. Liu, J. H. Zhao, Y. Y. Yang, Y. S. Liu and G. G. Ying, *Environ. Int.*, 2022, **163**, 107219.

20 Y. Li, J. Li, L. Zhang, Z. Huang, Y. Liu, N. Wu, J. He, Z. Zhang, Y. Zhang and Z. Niu, *Environ. Int.*, 2019, **123**, 87–95.

21 G. Lu, P. Shao, Y. Zheng, Y. Yang and N. Gai, *Int. J. Environ. Res. Public Health*, 2022, **19**, 5722.

22 Y. Hong, Q. Ding, T. Yang, X. Li, N. Song and J. Zhang, *Environ. Geochem. Health*, DOI:10.1007/S10653-025-02506-9.

23 Y. H. Jin, W. Liu, I. Sato, S. F. Nakayama, K. Sasaki, N. Saito and S. Tsuda, *Chemosphere*, 2009, **77**, 605–611.

24 S. Zhang, Q. Kang, H. Peng, M. Ding, F. Zhao, Y. Zhou, Z. Dong, H. Zhang, M. Yang, S. Tao and J. Hu, *Environ. Int.*, 2019, **126**, 54–60.

25 Y. Zhang, J. Meng, Y. Zhou, N. Song, Y. Zhao, M. Hong, J. Yu, L. Cao, Y. Dou and D. Kong, *Science of The Total Environment*, 2024, **920**, 171010.

26 L. Ahrens, S. Taniyasu, L. W. Y. Yeung, N. Yamashita, P. K. S. Lam and R. Ebinghaus, *Water Res.*, 2021, **198**, 117162.

27 M. Jurikova, D. Dvorakova and J. Pulkrabova, *Environmental Science and Pollution Research*, 2022, **29**, 60341–60353.

28 F. Kozisek, D. Dvorakova, F. Kotal, H. Jeligova, L. Mayerova, V. Svobodova, M. Jurikova, V. Gomersall and J. Pulkrabova, *Chemosphere*, 2025, **370**, 143969.

29 T. Teymoorian, L. Delon, G. Munoz and S. Sauvé, *Environ. Sci. Technol. Lett.*, 2025, **12**, 327–333.

30 V. Gellrich, H. Brunn and T. Stahl, *J. Environ. Sci. Health A Tox. Hazard. Subst. Environ. Eng.*, 2013, **48**, 129–135.

31 M. Llorca, M. Farré, Y. Picó, J. Müller, T. P. Knepper and D. Barceló, *Science of The Total Environment*, 2012, **431**, 139–150.

32 D. Skutlarek, M. Exner and H. Färber, *Environmental Science and Pollution Research*, 2006, **13**, 299–307.

33 V. Ingold, A. Kämpfe and A. S. Ruhl, *Eco-Environment & Health*, 2023, **2**, 235–242.

34 D. K. Essumang, A. Eshun, J. N. Hogarh, J. K. Bentum, J. K. Adjei, J. Negishi, S. Nakamichi, M. Habibullah-Al-Mamun and S. Masunaga, *Science of The Total Environment*, 2017, **579**, 729–735.

35 E. Zafeiraki, D. Costopoulou, I. Vassiliadou, L. Leondiadis, E. Dassenakis, W. Traag, R. L. A. P. Hoogenboom and S. P. J. van Leeuwen, *Food Addit. Contam. Part A Chem. Anal. Control Expo. Risk Assess.*, 2015, **32**, 2048–2057.

36 N. Li, G. G. Ying, H. Hong and W. J. Deng, *Environmental Pollution*, 2021, **270**, 116219.

37 Z. Manoochehri, B. Shoshtari-Yeganeh, L. Gheisari and K. Ebrahimpour, *J. Environ. Health Sci. Eng.*, DOI:10.1007/S40201-024-00930-0.

38 S. Harrad, N. Wemken, D. S. Drage, M. A. E. Abdallah and A. M. Coggins, *Environ. Sci. Technol.*, DOI:10.1021/ACS.EST.9B04604/SUPPL_FILE/ES9B04604_SI_001.PDF.

39 D. E. M. Haron, M. Yoneda, R. Hod, M. R. Ramli and M. Y. Aziz, *Environmental Science and Pollution Research*, 2023, **30**, 111062–111075.

40 S. H. Brandsma, J. C. Koekkoek, M. J. M. van Velzen and J. de Boer, *Chemosphere*, 2019, **220**, 493–500.

41 L. S. Haug, S. Salihovic, I. E. Jogsten, C. Thomsen, B. van Bavel, G. Lindström and G. Becher, *Chemosphere*, 2010, **80**, 1137–1143.

42 K. T. Tan, P. Shen, W. M. Ang, I. R. L. Lim, W. Z. Yu, Y. Wu and S. H. Chan, *ACS ES&T Water*, 2025, **5**, 4403.

43 H. Park, G. Choo, H. Kim and J. E. Oh, *Science of The Total Environment*, 2018, **634**, 1505–1512.

44 D. H. Kim, J. H. Lee and J. E. Oh, *J. Hazard. Mater.*, 2019, **365**, 26–33.

45 S. K. Kim, Y. L. Kho, M. Shoeib, K. S. Kim, K. R. Kim, J. E. Park and Y. S. Shin, *Environmental Pollution*, 2011, **159**, 1167–1173.

46 D. Cserbik, M. Casas, C. Flores, A. Paraian, L. S. Haug, I. Rivas, M. Bustamante, P. Dadvand, J. Sunyer, M. Vrijheid and C. M. Villanueva, *Journal of Exposure Science & Environmental Epidemiology 2023 34:1*, 2023, **34**, 90–96.

47 J. L. Domingo, I. Ericson-Jogsten, G. Perelló, M. Nadal, B. Van Bavel and A. Kärrman, *J. Agric. Food Chem.*, 2012, **60**, 4408–4415.

48 I. Ericson, M. Nadal, B. Van Bavel, G. Lindström and J. L. Domingo, *Environ. Sci. Pollut. Res. Int.*, 2008, **15**, 614–619.

49 T. Lertassavakorn, N. Pholphana, N. Rangkadilok, T. Suriyo and J. Satayavivad, *Food Addit. Contam. Part A Chem. Anal. Control Expo. Risk Assess.*, 2021, **38**, 1400–1415.

50 B. Ünlü Endirlik, E. Bakır, İ. İ. Boşgelmez, A. Eken, İ. Narin and A. Gürbay, *Chemosphere*, 2019, **235**, 1162–1171.

51 X. Li, M. Fatowe, D. Cui and N. Quinete, *Science of The Total Environment*, 2022, **806**, 150393.

52 C. S. Skaggs and B. A. Logue, *J. Chromatogr. A*, 2021, **1659**, 462493.

53 K. Dasu, S. F. Nakayama, M. Yoshikane, M. A. Mills, J. M. Wright and S. Ehrlich, *J. Chromatogr. A*, 2017, **1494**, 46–54.

54 N. J. Herkert, J. Merrill, C. Peters, D. Bollinger, S. Zhang, K. Hoffman, P. L. Ferguson, D. R. U. Knappe and H. M. Stapleton, *Environ. Sci. Technol. Lett.*, 2020, **7**, 178–184.

55 T. D. Sinkway, Q. Mehdi, E. K. Griffin, K. Correia, C. G. Camacho, J. Aufmuth, C. Ilvento and J. A. Bowden, *Science of The Total Environment*, 2024, **926**, 171932.

56 K. Hohweiler, L. A. Krometis, E. J. Ling and K. Xia, *Science of The Total Environment*, 2024, **929**, 172539.

57 J. Von Behren, P. Reynolds, P. M. Bradley, J. L. Gray, D. W. Kolpin, K. M. Romanok, K. L. Smalling, C. Carpenter, W. Avila, A. Ventura, P. B. English, R. R. Jones and G. M. Solomon, *Science of The Total Environment*, 2024, **953**, 176067.

58 S. M. Hall, S. Zhang, G. H. Tait, K. Hoffman, D. N. Collier, J. A. Hoppin and H. M. Stapleton, *Science of The Total Environment*, 2023, **895**, 165091.

59 X. Li, M. Fatowe, L. Lemos and N. Quinete, *Environ. Sci. Pollut. Res. Int.*, 2022, **29**, 84383–84395.

60 M. S. Alam, A. Abbasi and G. Chen, *Environ. Res.*, 2026, **294**, 123822.

61 N. H. Lam, C. R. Cho, K. Kannan and H. S. Cho, *J. Hazard. Mater.*, 2017, **323**, 116–127.
